# NINJ1 Facilitates Abdominal Aortic Aneurysm Formation via Blocking TLR4‐ANXA2 Interaction and Enhancing Macrophage Infiltration

**DOI:** 10.1002/advs.202306237

**Published:** 2024-06-23

**Authors:** Zhaoyu Wu, Zhijue Xu, Hongji Pu, Ang'ang Ding, Jiateng Hu, Jiahao Lei, Chenlin Zeng, Peng Qiu, Jinbao Qin, Xiaoyu Wu, Bo Li, Xin Wang, Xinwu Lu

**Affiliations:** ^1^ Department of Vascular Surgery Shanghai Ninth People's Hospital Shanghai JiaoTong University School of Medicine Shanghai 200011 China; ^2^ Vascular Center of Shanghai JiaoTong University Shanghai 200011 China; ^3^ Key Laboratory of Systems Biomedicine (Ministry of Education) Shanghai Center for Systems Biomedicine Shanghai Jiao Tong University Shanghai 200240 China; ^4^ Department of Ultrasound Shanghai Ninth People's Hospital Shanghai JiaoTong University School of Medicine Shanghai 200011 China

**Keywords:** abdominal aortic aneurysm, infiltration, inflammation, macrophage, NINJ1

## Abstract

Abdominal aortic aneurysm (AAA) is a common and potentially life‐threatening condition. Chronic aortic inflammation is closely associated with the pathogenesis of AAA. Nerve injury‐induced protein 1 (NINJ1) is increasingly acknowledged as a significant regulator of the inflammatory process. However, the precise involvement of NINJ1 in AAA formation remains largely unexplored. The present study finds that the expression level of NINJ1 is elevated, along with the specific expression level in macrophages within human and angiotensin II (Ang II)‐induced murine AAA lesions. Furthermore, *Ninj1^flox/flox^* and *Ninj1^flox/flox^Lyz2‐Cre* mice on an *ApoE^−/−^* background are generated, and macrophage NINJ1 deficiency inhibits AAA formation and reduces macrophage infiltration in mice infused with Ang II. Consistently, in vitro suppressing the expression level of NINJ1 in macrophages significantly restricts macrophage adhesion and migration, while attenuating macrophage pro‐inflammatory responses. Bulk RNA‐sequencing and pathway analysis uncover that NINJ1 can modulate macrophage infiltration through the TLR4/NF‐κB/CCR2 signaling pathway. Protein‐protein interaction analysis indicates that NINJ1 can activate TLR4 by competitively binding with ANXA2, an inhibitory interacting protein of TLR4. These findings reveal that NINJ1 can modulate AAA formation by promoting macrophage infiltration and pro‐inflammatory responses, highlighting the potential of NINJ1 as a therapeutic target for AAA.

## Graphical abstract

NINJ1 is highly expressed in macrophages within human and murine abdominal aortic aneurysm (AAA) lesions, which enhances macrophage infiltration through the TLR4/NF‐κB/CCR2 signaling pathway, thus facilitating AAA formation. NINJ1 activates TLR4 by competitively binding with ANXA2, an inhibitory interacting protein of TLR4. This study highlights the potential of NINJ1 as a therapeutic target for AAA.

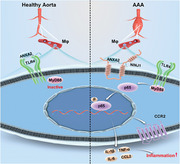

## 1 Introduction

Abdominal aortic aneurysm (AAA) is a common and catastrophic aortic disease with high rates of fatality and disability, causing more than 150 thousand deaths every year worldwide.^[^ ^1^, ^2^ ^]^ The major limitation of the clinical management of AAA is the lack of appropriate treatment modalities to restrict the growth and rupture of aneurysms.^[^ ^3^, ^4^ ^]^ Therefore, there is an urgent need to understand the pathogenesis and to find therapeutic targets for AAA. A hallmark of AAA is the prominent inflammatory cell infiltration.^[^ ^5^ ^]^ Previous studies have revealed that chronic aortic inflammation leads to loss of vascular smooth muscle cells (VSMCs) and destruction of the aortic wall.^[^ ^6^, ^7^ ^]^ Nevertheless, the underlying mechanisms of aortic inflammation in the development and progression of AAA should be elucidated.

Clinical trials have demonstrated that the level of macrophage‐mediated inflammation is a definite prognostic factor for the growth, rupture, and repair of AAA,^[^ ^8^, ^9^, ^10^, ^11^ ^]^ emphasizing the importance of macrophages in the pathological process of AAA. Macrophages infiltrate the aortic wall from periadventitial lymph nodes or through adventitial vasa vasorum, and they also enhance the inflammatory response by producing cytokines.^[^ ^12^, ^13^ ^]^ Moreover, macrophages generate substantial proteases and oxygen‐derived free radicals to induce apoptosis of VSMCs and destruction of the aortic wall.^[^ ^14^, ^15^ ^]^ Our previous study indicated that inhibition of inflammation in macrophages can partially alleviate the formation and expansion of AAA.^[^ ^16^ ^]^ However, the regulatory mechanisms of infiltration and activation of macrophages in AAA have not been fully explored.

Nerve injury‐induced protein 1 (NINJ1), a transmembrane protein that is highly expressed by macrophages, has been proven to be involved in the regulation of several immune‐related inflammatory processes.^[^ ^17^, ^18^, ^19^, ^20^, ^21^, ^22^ ^]^ NINJ1 can promote adhesion and migration of macrophages across blood‐brain barrier endothelial cells, thereby enhancing the inflammation of the central nervous system.^[^ ^19^, ^23^, ^24^ ^]^ NINJ1 also upregulates the expression levels and secretion of pro‐inflammatory cytokines in macrophages,^[^ ^18^, ^25^ ^]^ via modulating the Toll‐like receptor signaling pathway.^[^ ^26^ ^]^ Recently, NINJ1 was found to mediate plasma membrane rupture and subsequent release of damage‐associated molecular patterns, resulting in the amplification of the inflammatory response.^[^ ^27^, ^28^ ^]^ NINJ1 has been reported to be closely associated with atherosclerosis and vascular dysfunctions;^[^ ^29^, ^30^, ^31^, ^32^, ^33^ ^]^ However, the functional role of NINJ1 in AAA has still remained elusive.

The present study aimed to delineate the roles and underlying mechanisms of NINJ1 during AAA formation. A significant upregulation of NINJ1 in both human and murine AAA lesions, particularly in macrophages, was found. To investigate the contribution of macrophage‐derived NINJ1 to AAA formation, it was attempted to generate the conditional macrophage‐specific *Ninj1* knockout mice, and it was demonstrated that macrophage‐derived NINJ1 promoted AAA formation by enhancing macrophage infiltration and inflammation. Furthermore, the findings revealed that NINJ1 activated the TLR4/NF‐κB/CCR2 pathway through its interaction with ANXA2, leading to the release of TLR4 from the inhibitory interaction between ANXA2 and TLR4.

## 2 Results

### 2.1 NINJ1 was Elevated in AAA Tissues, Particularly in Macrophages

**Figure 1:**
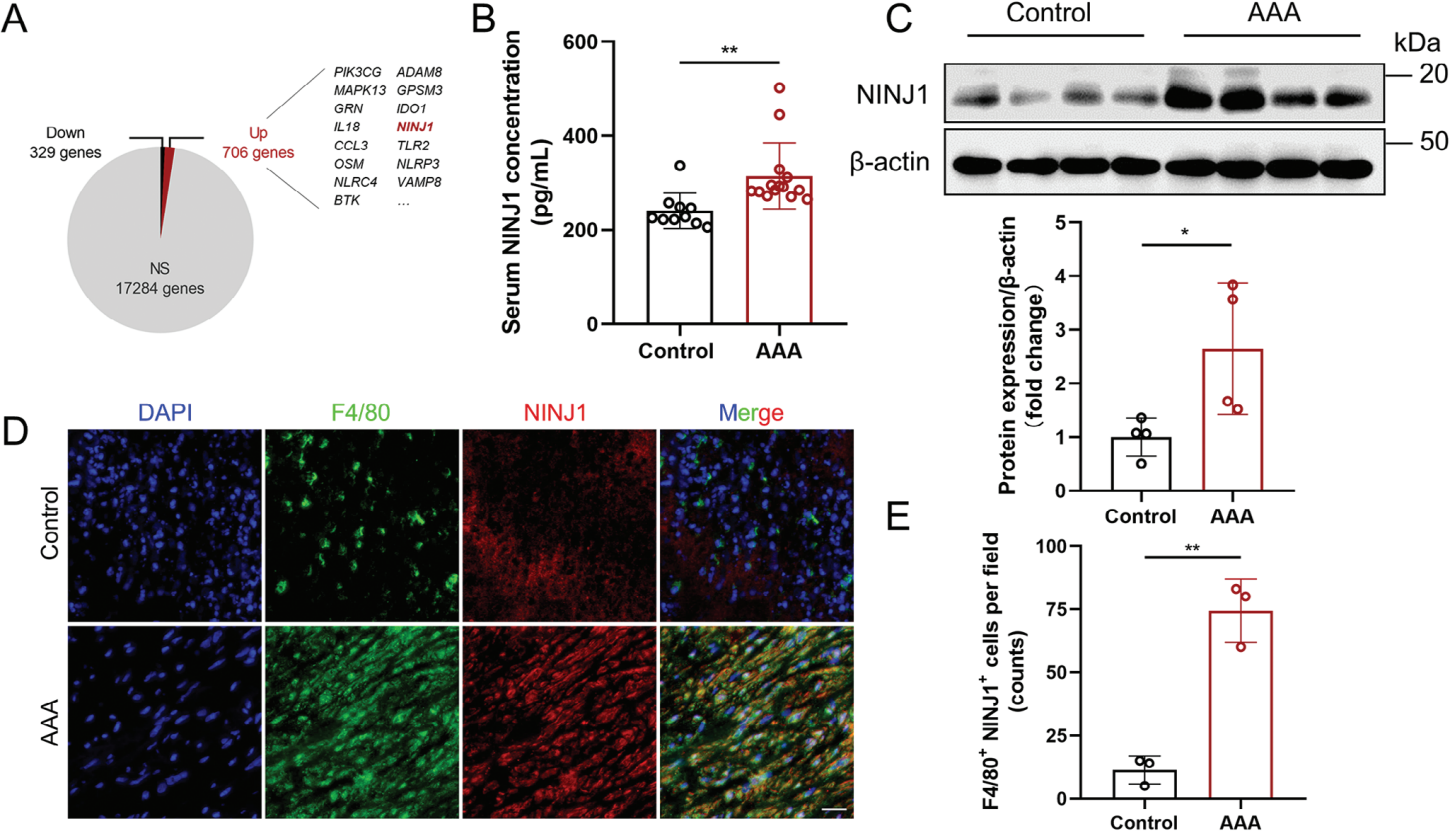
NINJ1 is upregulated in abdominal aortic aneurysm (AAA) tissues, particularly in macrophages. A) The pie chart presents the upregulated differentially expressed genes (DEGs) associated with the positive regulation of the inflammatory response pathway (GO: 00 50729), ordered by increasing *P*‐values. DEGs were identified based on |log2(fold change [FC])| ≥ 1 and *p‐*value < 0.05. NS indicates *p *> 0.05. B) Serum samples from AAA patients (*n* = 14) and control individuals (*n* = 10) were analyzed using ELISA to measure NINJ1 protein concentration. Statistical analysis was performed using the Student's *t*‐test. ^**^ *p* < 0.01. C) Western blot analysis was used to determine NINJ1 protein levels in murine AAA and normal abdominal aortic tissues. β‐actin protein levels were used for normalization. Statistical analysis was performed using the Student's *t*‐test; *n* = 4. ^*^ *p *< 0.05. D) Representative immunofluorescent images of NINJ1 (red) expression in human AAA and non‐AAA tissues, along with co‐staining for the macrophage‐associated marker F4/80 (green) and DAPI (blue). Scale bar = 20 µm. E) Quantification of double‐labeled cells expressing both F4/80 and NINJ1 in panel D. Statistical analysis was performed using the Student's *t*‐test; *n* = 3. ^**^ *p *< 0.01.

To explore novel clinically relevant genes with potential for the formation and progression of AAA, a microarray‐based gene expression dataset of human abdominal aortic tissues (GEO database, GSE7084) was analyzed, and the inflammation‐related genes were assessed, considering the important role of inflammation in AAA pathogenesis. Among the 706 unexpressed genes, 33 were enriched in the positive regulation of inflammatory response pathway (GO: 00 50729), with *NINJ1* emerging as one of the most significantly altered genes, exhibiting a 2.35‐fold change compared with the normal abdominal aorta tissues (**Figure** **1**A; Figure S1A–C, Supporting Information). To verify the clinical relevance of NINJ1 and AAA, the expression level of NINJ1 was analyzed in human samples and murine models of Ang II‐induced AAA. The results of ELISA indicated a higher serum NINJ1 concentration in the AAA group compared with that in the non‐AAA group in clinical samples (Figure 1B), but no significant difference in serum NINJ1 concentration was found between control and angiotensin II (Ang II)‐induced AAA mice (Figure S1D, Supporting Information). Immunoblotting of murine AAA tissues and immunofluorescence staining of human AAA tissues confirmed the upregulation of NINJ1 expression level in AAA lesions (Figure 1C–E). Furthermore, single‐cell RNA sequencing (scRNA‐seq) analysis indicated that *Ninj1* expression was markedly elevated in the macrophage cluster within murine AAA tissues in comparison to control samples (Figure S1E–I, Supporting Information). Immunofluorescence staining further confirmed that NINJ1 was predominantly located in macrophages in human AAA tissues (Figure 1D,E, Supporting Information), as well as in the murine AAA model (Figure S1J–O, Supporting Information). Taken together, these results highlighted the upregulation of NINJ1 expression levels in AAA lesions, particularly in macrophages.

### 2.2 Macrophage NINJ1 Deficiency Attenuates Ang II‐Induced AAA Formation

To elucidate the role of macrophage‐derived NINJ1 in AAA formation and progression, *Ninj1^flox/flox^* mice were crossed with mice transgenically expressing Cre from the myeloid cell‐specific gene encoding LysM (*Lyz2‐Cre*) on an *ApoE* knockout background to generate macrophage NINJ1‐deficient *ApoE* ^−/−^ mice (*ApoE* ^−/−^ *Ninj1^flox/flox^Lyz2‐Cre*), hereafter referred to as *Ninj1^ΔMΦ^* mice (Figure S2A–C, Supporting Information). The mRNA expression levels of *Ninj1* in bone marrow‐derived macrophages (BMDMs) and spleen tissues from *Ninj1^ΔMΦ^* mice were significantly reduced compared with those in the *ApoE* ^−/−^ *Ninj1^flox/flox^* group (hereafter referred to as *Ninj1^fl/fl^* mice), verifying the deficiency of macrophage‐derived NINJ1 (Figure S2D,E, Supporting Information). The effects of macrophage NINJ1 deficiency on the sex ratio, diet, hair, and weight were assessed, and no significant difference was found between *Ninj1^ΔMΦ^* and *Ninj1^fl/fl^* mice (Figure S2F, Supporting Information).

**Figure 2:**
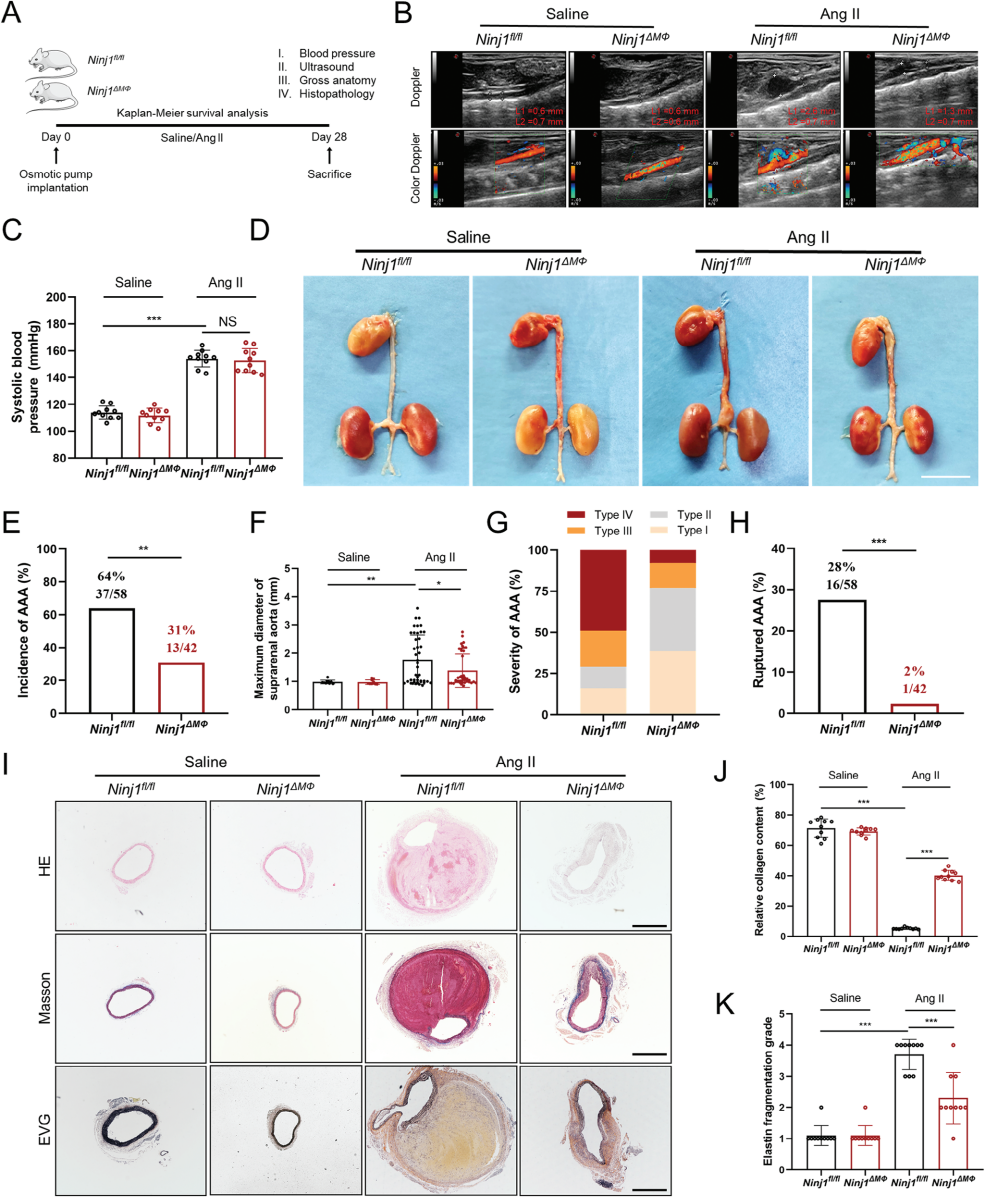
Macrophage NINJ1 deficiency attenuates angiotensin II (Ang II)‐induced abdominal aortic aneurysm (AAA) formation. A) Schematic protocol for the establishment of a murine model of AAA. B) Representative ultrasound and color Doppler ultrasound images of the abdominal aorta of *ApoE* ^−/−^ *Ninj1^flox/flox^* (*Ninj1^fl/fl^*) and *ApoE* ^−/−^ *Ninj1^flox/flox^Lyz2‐Cre* (*Ninj1^ΔMΦ^*) mice infused with saline or Ang II for 4 weeks. C) The systolic blood pressure (SBP) of *Ninj1^fl/fl^* and *Ninj1^ΔMΦ^* mice infused with saline or Ang II for 4 weeks. Data were analyzed by two‐way ANOVA, followed by the Bonferroni post hoc test; *n* = 10 mice per group. ^***^ *p *< 0.001; NS indicates *p *> 0.05. D) Representative macroscopic images of AAA formation in the indicated groups. Scale bar = 1 cm. E) The incidence of AAA in *Ninj1^fl/fl^* (*n* = 58) and *Ninj1^ΔMΦ^* (*n* = 42) mice infused with Ang II for 4 weeks. Data were analyzed by Fisher's exact test. ^**^ *p *< 0.01. F) The maximum diameter of the abdominal aorta in the indicated groups. Data were analyzed by 2‐way ANOVA followed by the Bonferroni post hoc test. ^*^ *p *< 0.05; ^**^ *p *< 0.01. G) The classification of severity of AAA in *Ninj1^fl/fl^* and *Ninj1^ΔMΦ^* mice infused with Ang II for 4 weeks. H) The rupture rate of AAA in *Ninj1^fl/fl^* and *Ninj1^ΔMΦ^* mice infused with Ang II for 4 weeks. Data were analyzed by Fisher's exact test. ^***^ *p *< 0.001. I) The representative images of hematoxylin‐eosin (HE), Masson, and Elastic Van Gieson (EVG) staining of abdominal aorta in the indicated groups. Scale bar = 500 µm. J) Quantification of collagen content in murine abdominal aortic tissues in panel I. Data were analyzed by two‐way ANOVA followed by the Bonferroni post hoc test; *n* = 10 mice per group. ^***^ *p *< 0.001. K) Elastin degradation grade of murine abdominal aortic tissues in panel I. Data were analyzed by two‐way ANOVA followed by the Bonferroni post hoc test; *n* = 10 mice per group. ^***^ *p *< 0.001.

Subsequently, *Ninj1^ΔMΦ^* and *Ninj1^fl/fl^* mice were subjected to the Ang II‐induced AAA model (**Figure** **2**A). On day 28 after Ang II infusion, the abdominal diameters were detected by ultrasonography, and the murine systolic blood pressure (SBP) was measured using a noninvasive tail‐cuff system. The aortic diameters of *Ninj1^ΔMΦ^* mice were significantly lower than those of *Ninj1^fl/fl^* mice (Figure 2B), while the SBP showed no significant difference between the 2 groups (Figure 2C), suggesting that macrophage NINJ1 deficiency reduced dilatation of abdominal aortic with no effect on SBP. From gross anatomical inspection, *Ninj1^ΔMΦ^* mice developed smaller AAAs compared with their *Ninj1^fl/fl^* counterparts (Figure 2D). The incidence of AAA was reduced from 64% (37 of 58) in *Ninj1^fl/fl^* mice to 31% (13 of 42) in *Ninj1^ΔMΦ^* mice (Figure 2E). Consistently, the maximum aortic diameter and the severity of AAA were attenuated by macrophage NINJ1 deficiency (Figure 2F,G). Notably, after 28 days of Ang II infusion, *Ninj1^fl/fl^* mice revealed a higher rupture rate of AAA, which was markedly reduced in *Ninj1^ΔMΦ^* mice (28% vs 2%, *p *< 0.001, Figure 2H). Masson and Elastic Van Gieson (EVG) staining of the AAA lesions exhibited that the reduction of collagen content and the fragmentation of elastic fibers in *Ninj1^ΔMΦ^* mice treated with Ang II were markedly lower than those in *Ninj1^fl/fl^* mice (Figure 2I–K), indicating that macrophage NINJ1 deficiency inhibited the degradation of collagen and elastic fibers. Moreover, TdT‐mediated dUTP nick‐end labeling (TUNEL) staining and immunohistochemistry indicated that the levels of cell apoptosis, pyroptosis, and necrosis in the abdominal aorta wall were reduced in *Ninj1^ΔMΦ^* mice (Figure S3A–D, Supporting Information). Collectively, these results indicated that the deficiency of NINJ1 in macrophages attenuated the formation of Ang II‐induced AAA.

### 2.3 Macrophage NINJ1 Deficiency Repressed Inflammation of Abdominal Aortic Wall in AAA Formation

**Figure 3:**
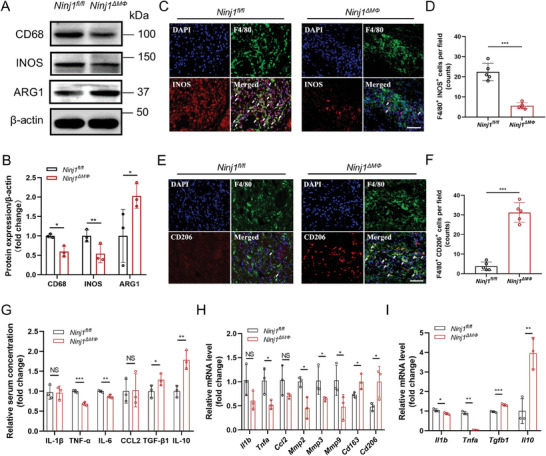
Macrophage NINJ1 deficiency inhibits inflammation of the abdominal aortic wall in AAA formation A) The levels of CD68, INOS, and ARG1 proteins in murine AAA tissues were measured by Western blotting. B) Quantification of the expression levels of CD68, INOS, and ARG1 measured by Western blotting in panel A. The level of β‐actin protein was used for normalization. Data were analyzed by the Student's *t*‐test; *n* = 3 independent experiments. ^*^ *p *< 0.05; ^**^ *p *< 0.01. C) and D) Representative images of INOS (red) expression level by immunofluorescence staining of murine AAA tissues, and co‐staining with the macrophage‐associated marker F4/80 (green) and DAPI (blue). White arrows indicate F4/80^+^/ INOS^+^ cells. Scale bar = 50 µm. Data were analyzed by the Student's *t*‐test; *n* = 5. ^***^ *p *< 0.001. E) and F) Representative images of CD206 (red) expression level by immunofluorescence staining of murine AAA tissues, and co‐staining with the macrophage‐associated marker F4/80 (green) and DAPI (blue). White arrows indicate F4/80^+^/ CD206^+^ cells. Scale bar = 50 µm. Data were analyzed by the Student's *t*‐test; *n* = 5. ***P < 0.001. (G) ELISA of IL‐1β, TNF‐α, IL‐6, CCL2, TGF‐β1, and IL‐10 concentrations in serum samples from *Ninj1^fl/fl^* and *Ninj1^ΔMΦ^* mice. Data were analyzed by the Student's *t*‐test; *n* = 3. NS indicates *p *> 0.05; ^*^ *p *< 0.05; ^**^ *p *< 0.01; ^***^ *p *< 0.001. H) Quantitative polymerase chain reaction (qPCR) analysis of *Il1b*, *Tnfa*, *Ccl2*, *Mmp2*, *Mmp3*, *Mmp9*, *Cd163*, and *Cd206* mRNA expression levels in spleen tissues in the indicated groups. Data were analyzed by the Student's *t*‐test; *n* = 3. NS indicates *p *> 0.05; ^*^ *p *< 0.05. (I) Quantification of *Il1b*, *Tnfa*, *Tgfb1*, and *Il10* mRNA expression levels by quantitative PCR (qPCR) in murine bone marrow‐derived macrophages (BMDMs). Data were analyzed by the Student's *t*‐test; *n* = 3. ^*^ *p *< 0.05; ^**^ *p *< 0.01; ^***^ *p *< 0.001.

Macrophage‐mediated inflammation process plays a critical role in the initiation and progression of the aneurysmal process.^[^ ^13^ ^]^ In the abdominal aorta of *Ninj1^ΔMΦ^* mice treated with Ang II, the expression levels of macrophage–associated marker (CD68) and pro‐inflammatory marker (INOS) were downregulated compared with that of *Ninj1^fl/fl^* mice, while the expression level of anti‐inflammatory marker (ARG1) was upregulated (**Figure** **3**A,B). Consistently, in situ immunofluorescence staining exhibited that macrophages in AAA lesions from *Ninj1^ΔMΦ^* mice showed a reduced expression level of INOS and the enhanced expression level of CD206 (Figure 3C–F), indicating that macrophage NINJ1 deficiency inhibited pro‐inflammatory phenotype of macrophages in AAA. The levels of inflammation‐related cytokines in murine serum and spleen tissues were then evaluated. The results of ELISA assays indicated that the expression levels of pro‐inflammatory cytokines (TNF‐α and IL‐6) were significantly reduced in serum from *Ninj1^ΔMΦ^* mice, while the expression levels of anti‐inflammatory cytokines (TGF‐β1 and IL‐10) were markedly elevated (Figure 3G). The RT‐qPCR performed on murine spleen tissues revealed that the mRNA expression levels of pro‐inflammatory markers (*Tnfa*, *Mmp2*, *Mmp3*, and *Mmp9*) were downregulated in the *Ninj1^ΔMΦ^* group compared with those in the *Ninj1^fl/fl^* group, which is consistent with the results of immunohistochemical staining (Figure 3H; Figure S4A–D, Supporting Information), whereas the mRNA levels of anti‐inflammatory markers (*Cd163* and *Cd206*) were upregulated (Figure 3H). Furthermore, BMDMs from *Ninj1^ΔMΦ^* mice exhibited reduced expression of pro‐inflammatory genes (*Il1b* and *Tnfa*) and rescued expression of anti‐inflammatory genes (*Tgfb1* and *Il10*) compared with those from *Ninj1^fl/fl^* mice (Figure 3I). Collectively, these results demonstrated that deficiency of NINJ1 reduced macrophage‐mediated inflammation in AAA formation.

### 2.4 NINJ1 Modulated Inflammation and Infiltration Pathways in Macrophages

**Figure 4:**
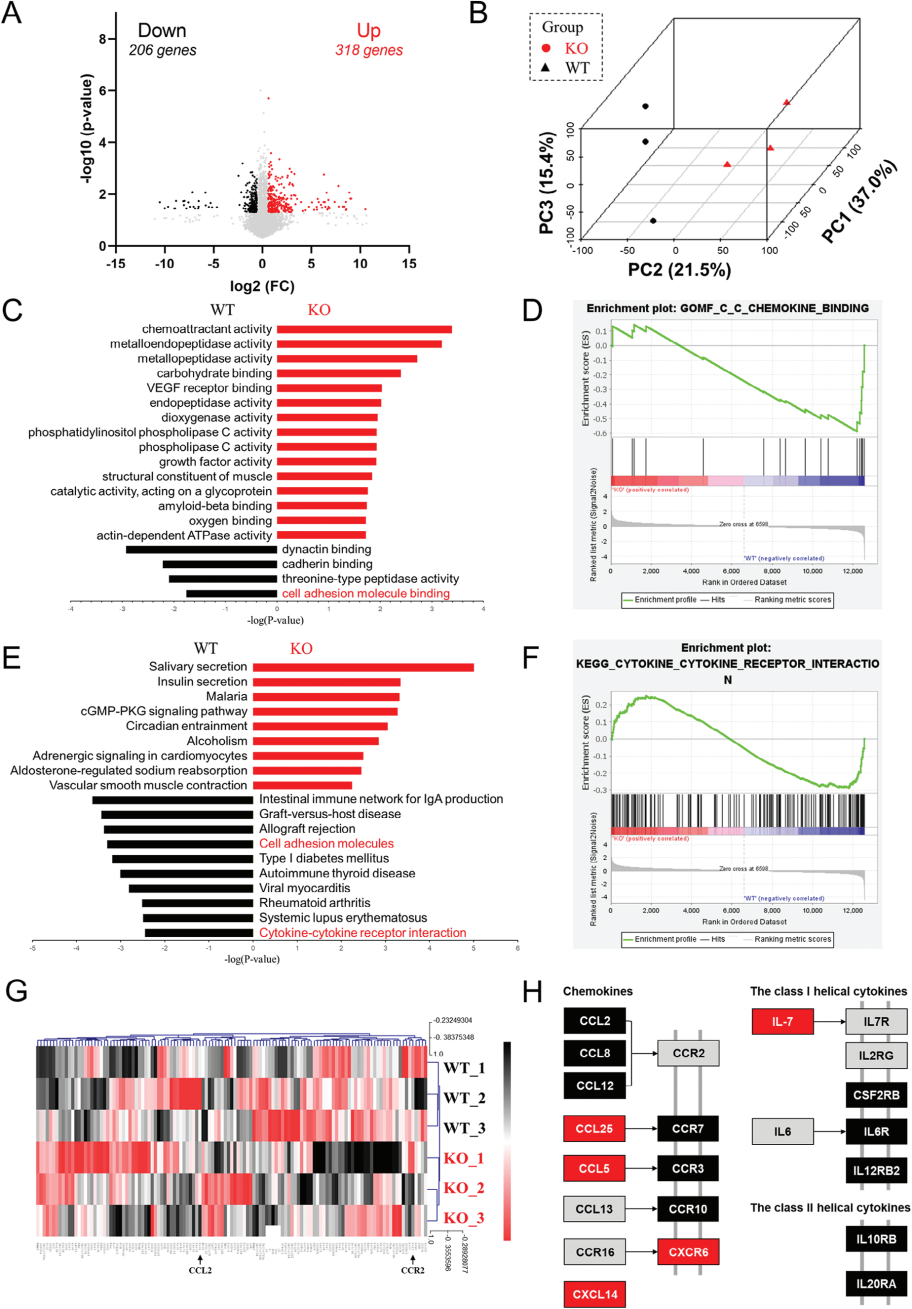
NINJ1 modulates inflammation and infiltration pathways in macrophages. A) Volcano plot of differentially expressed genes (DEGs) between bone marrow‐derived macrophages (BMDMs) from *Ninj1^fl/fl^* (WT) and *Ninj1^ΔMΦ^* (KO) mice; *n* = 3 mice per group. DEGs were defined as genes with a fold change (FC) ≥ 1.50 or ≤ 0.67 and *p *< 0.05. Upregulated genes were displayed in red, and downregulated genes were shown in black. B) Principal component analysis plot of the RNA‐sequencing data for the indicated groups. C) Clustering of enriched Gene Ontology (GO)‐molecular function (MF) pathways for DEGs. D) The C‐C chemokine binding pathway (GO: 00 19957) was significantly downregulated in BMDMs from *Ninj1^ΔMΦ^* mice. E) Clustering of enriched Kyoto Encyclopedia of Genes and Genomes (KEGG) pathways for DEGs. F) The cytokine‐cytokine receptor interaction pathway (KEGG: mmu04060) was significantly downregulated in BMDMs from *Ninj1^ΔMΦ^* mice. G) Heatmap of gene expression of cytokine‐cytokine receptor interaction pathway in the indicated groups. H) The KEGG pathway map of cytokine‐cytokine receptor interaction. The intact KEGG map is shown in Figure S5G (Supporting Information).

To obtain a deeper insight into the potential roles and mechanisms of NINJ1 in AAA formation, RNA‐seq of murine BMDMs from *Ninj1^ΔMΦ^* and *Ninj1^fl/fl^* mice was conducted. A total of 524 differentially expressed genes (DEGs) were identified in the 2 groups, of which 318 were upregulated in the *Ninj1^ΔMΦ^* group and 206 were downregulated (**Figure** **4**A,B). The Gene Ontology (GO) enrichment analysis of DEGs indicated that the downregulated DEGs were enriched in cell adhesion pathways (including dynactin binding, cadherin binding, and cell adhesion molecule binding) and inflammation pathways (negative regulation of interleukin 12 production and Toll‐like receptor 9 pathway) (Figure 4C,D; Figure S5A,B, Supporting Information). It is consistent with the Reactome analysis, in which the downregulated genes showed enrichment in pathways related to interferon‐γ and chemokine receptors bind chemokines (Figure S5C,D, Supporting Information). The Kyoto Encyclopedia of Genes and Genomes (KEGG) pathway analysis further revealed downregulation in pathways associated with cell adhesion molecules, inflammatory and immune diseases (including graft‐versus‐host disease, type I diabetes mellitus and autoimmune thyroid disease, etc), and cytokine‐cytokine receptor interaction (Figure 4E,F; Figure S5E,F, Supporting Information). Additionally, DEGs were identified in the cytokine‐cytokine receptor interaction pathway (Figure 4G), with 22 cytokines or receptors downregulated and 7 upregulated in BMDMs from *Ninj1^ΔMΦ^* mice (Figure 4H; Figure S5G, Supporting Information). These findings suggested that NINJ1 deficiency could lead to downregulation of the pro‐inflammatory phenotype and reduced infiltration capacity of macrophages.

To determine the role of NINJ1 in the inflammatory phenotype of macrophages, the NINJ1 overexpression and knockdown murine Raw264.7 macrophages were constructed, and they were verified by immunoblotting and RT‐qPCR (Figure S6A–E, Supporting Information). Subsequently, the expression levels of pro‐inflammatory factors (e.g., IL‐1β, TNF‐α, and IL‐6) and anti‐inflammatory factors (e.g., IL‐10, TGF‐β1, and ARG1) were analyzed by immunoblotting and RT‐qPCR. Overexpression of NINJ1 increased the expression levels of IL‐1β, TNF‐α, and IL‐6, while decreasing the expression levels of IL‐10, TGF‐β1, and ARG1 (Figure S6A,B,F, Supporting Information). These correlations of expression patterns were further confirmed in the NINJ1 knockdown groups (Figure S6A,C,G, Supporting Information). Moreover, ELISA revealed that the expression levels of IL‐1β, TNF‐α, IL‐6, and CCL2 were upregulated in cell supernatant of NINJ1 overexpression cells (Figure S6H–K, Supporting Information), while they were downregulated in cell supernatant of NINJ1 knockdown cells (Figure S6L–O, Supporting Information). Taken together, the results obtained from the NINJ1 overexpression and knockdown experiments provided evidence supporting the role of NINJ1 in promoting a pro‐inflammatory phenotype and the secretion of pro‐inflammatory cytokines in macrophages

### 2.5 NINJ1 Enhanced Adhesion and Trans‐endothelial Migration of Macrophages

**Figure 5:**
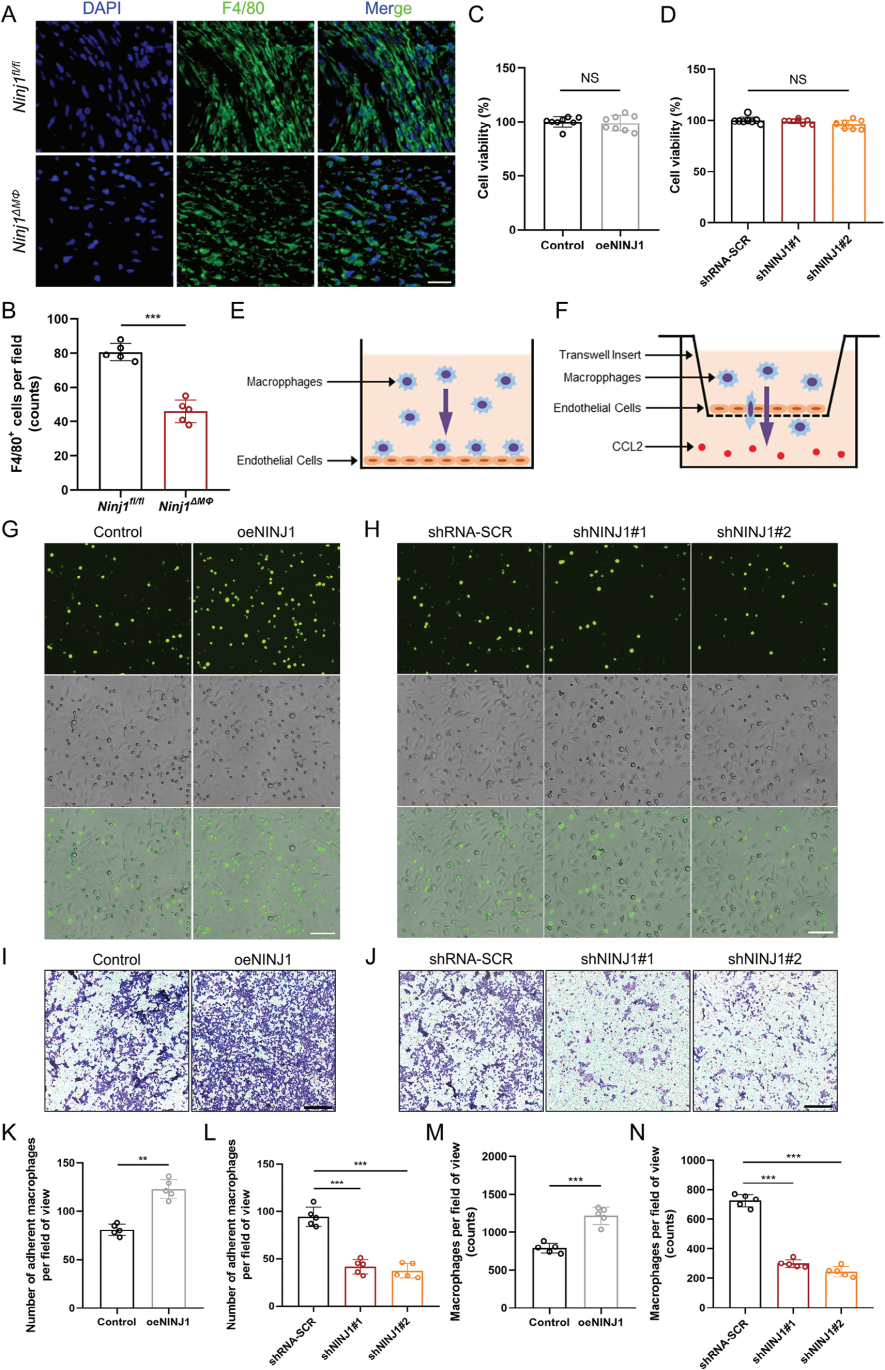
NINJ1 enhances the adhesion and trans‐endothelial migration of macrophages. A) and B) Representative images of F4/80 (green) expression level by immunofluorescence staining of murine AAA tissues (*n* = 5 per group), and co‐staining with DAPI (blue). Scale bar = 25 µm. Data were analyzed by the Student's *t*‐test; *n* = 5. ^***^ *p *< 0.001. C) Viability of NINJ1 overexpression Raw264.7 cells measured by the cell counting kit‐8 (CCK‐8) assay at 24 h after transfection. Data were analyzed by the Student's *t*‐test; *n* = 8. NS indicates *p *> 0.05. D) Viability of NINJ1 knockdown Raw264.7 cells measured by the CCK‐8 assay at 24 h after transfection. Data were analyzed by two‐way ANOVA, followed by the Bonferroni post hoc test; *n* = 8. NS indicates *p *> 0.05. E) Schematic diagram of cell adhesion assay. Raw264.7 cells were seeded into the 6‐well plates containing endothelial cells and were cocultured for 30 min, and suspended cells were thrice rinsed with PBS. F) Schematic diagram of transwell migration assay. Raw264.7 cells were seeded into the top chamber containing endothelial cells and were cocultured for 24 h. CCL2 was added into the lower chamber to induce trans‐endothelial migration. G) and H) Representative images of cell adhesion assay in the indicated groups. Raw264.7 cells were pre‐stained with fluorescent probe BCECF‐AM (green). Scale bar = 10 µm. I) and J) Representative images of transwell migration assay in the indicated groups. Scale bar = 200 µm. Raw264.7 cells were stained with crystal violet. K) Quantification of adherent Raw264.7 cells in panel G. Data were analyzed by Student's *t*‐test; *n* = 5. ^**^ *p *< 0.01.L) Quantification of adherent Raw264.7 cells in panel H. Data were analyzed by Student's *t*‐test; *n* = 5. ^***^ *p *< 0.001. M) Quantification of cell trans‐endothelial migration assay in panel I. Data were analyzed by the Student's *t*‐test; *n* = 5. ^***^ *p *< 0.001. N) Quantification of cell trans‐endothelial migration assay in panel J. Data were analyzed by the Student's *t*‐test; *n* = 5. ^***^ *p *< 0.001.

In the AAA lesions of *Ninj1^ΔMΦ^* mice, the number of macrophages was significantly less than that in *Ninj1^fl/fl^* mice (**Figure** **5**A,B), while overexpression and knockdown of NINJ1 showed no effect on the cell viability (Figure 5C,D). It suggested that deficiency of NINJ1 reduced the infiltration of macrophages, which was consistent with the results of the GO and KEGG pathway enrichment analyses of RNA‐sequencing (Figure 4C,E). Macrophage infiltration is a typical pathological change of the aortic wall in AAA.^[^ ^34^ ^]^ To examine the impact of NINJ1 on the cell adhesion and migration abilities, cell adhesion and transwell migration assays were carried out on the NINJ1 overexpression and knockdown Raw264.7 cells (Figure 5E,F). A substantial increase in macrophage adhesion was found in the NINJ1 overexpressed group (Figure 5G,K), which was significantly reduced by the knockdown of NINJ1 (Figure 5H,L). Moreover, macrophage trans‐endothelial migration was enhanced by overexpression of NINJ1 (Figure 5I,M), while it was attenuated by knockdown of NINJ1 (Figure 5J,N). These results indicated that NINJ1 enhanced the cell adhesion and trans‐endothelial migration of macrophages.

### 2.6 NINJ1 Modulates Macrophage Infiltration via Activating the TLR4/NF‐κB/CCR2 Pathway

**Figure 6:**
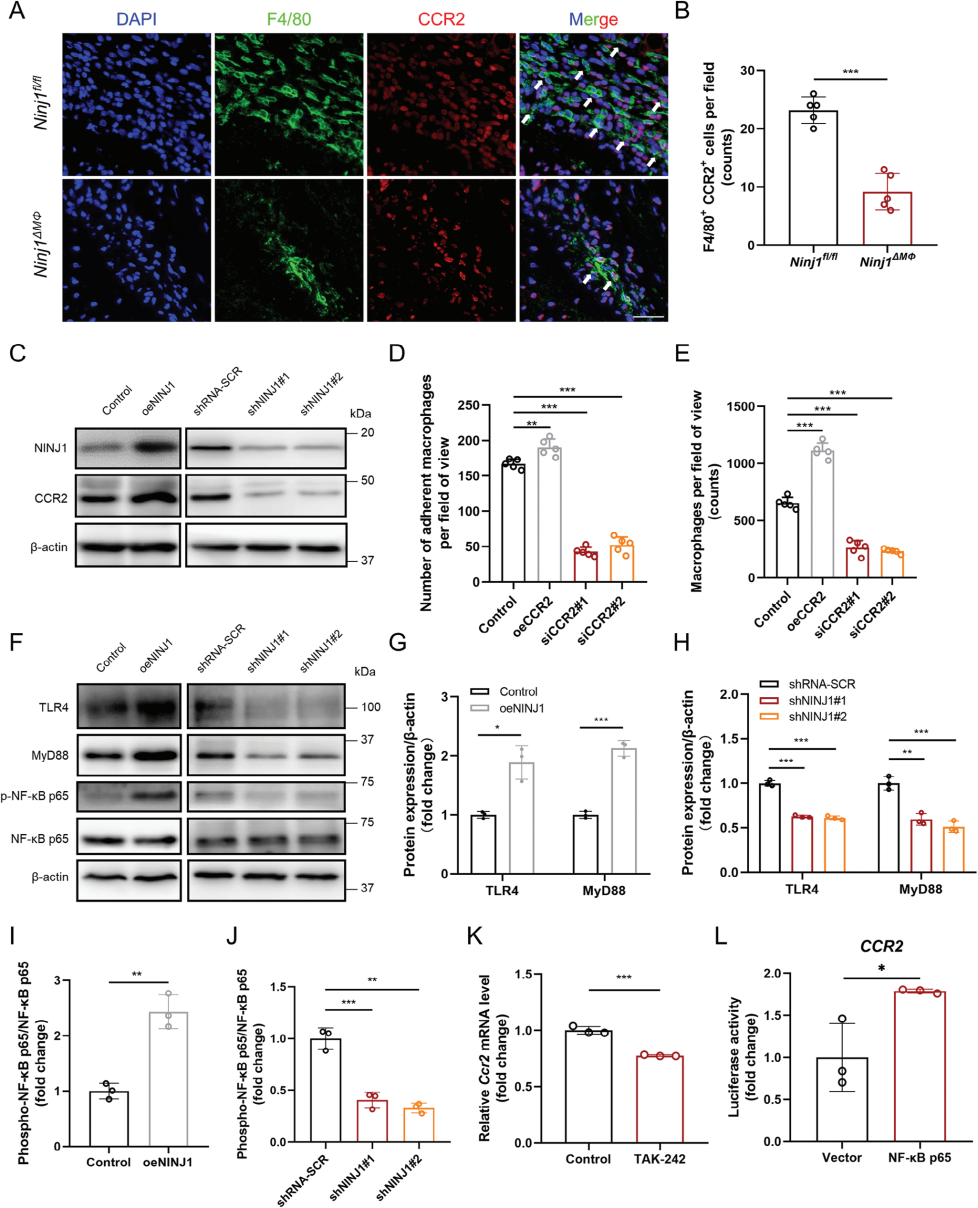
NINJ1 modulates macrophage infiltration by activating the TLR4/NF‐κB/CCR2 pathway. A) and B) Representative images of CCR2 (red) expression level by immunofluorescence staining of murine AAA tissues, and co‐staining with macrophage‐associated marker F4/80 (green) and DAPI (blue). White arrows indicate F4/80^+^/ CCR2^+^ cells. Scale bar = 25 µm. Data were analyzed by the Student's *t*‐test; *n* = 5. ^***^ *p *< 0.001. C) The levels of NINJ1 and CCR2 proteins were measured by Western blotting in the indicated groups; *n* = 3. D) Quantification of cell adhesion assay in the indicated groups. Data were analyzed by the Student's *t*‐test; *n* = 5. ^**^ *p *< 0.01; ^***^ *p *<  0.001. E) Quantification of cell trans‐endothelial migration assay in the indicated groups. Data were analyzed by the Student's *t*‐test; *n* = 5. ^***^ *p *< 0.001. F) The expression levels of TLR4, MyD88, phospho‐NF‐κB p65, and NF‐κB p65 proteins were measured by Western blotting in the indicated groups. G) and H) Quantification of the expression levels of TLR4 and MyD88 protein measured by Western blotting in panel F. The level of β‐actin protein was used for normalization. Data were analyzed by the Student's *t*‐test; *n* = 3. ^*^ *p *< 0.05; ^**^ *p *< 0.01; ^***^ *p *< 0.001. I) and J) Quantification of phosphorylation of NF‐κB p65 in panel F. Data were analyzed by the Student's *t*‐test; *n* = 3. **P < 0.01; ^***^ *p *< 0.001. K) Quantification of *Ccr2* mRNA expression level by quantitative polymerase chain reaction (PCR) in the indicated groups. Raw264.7 cells were treated with 1 µM TAK‐242 for 4 h. Data were analyzed by the Student's *t*‐test; *n* = 3. ^***^ *p *< 0.001. L) *CCR2* luciferase gene reporter activity in the indicated groups. HEK293T cells were co‐transfected with psiCHECK‐2 luciferase reporter vector containing human *CCR2* gene promotor and NF‐κB p65‐coding vector. Data were analyzed by the Student's *t*‐test; *n* = 3. ^*^ *p *< 0.05.

In the RNA‐sequencing analysis results mentioned above, the *Ninj1^ΔMΦ^* group showed enrichment of CCR2 and its ligands, including CCL2, CCL8, and CCL12, based on the KEGG pathway analysis (Figure 4H). Notably, CCR2 was found as one of the essential regulators for macrophage infiltration.^[^ ^35^ ^]^ It was identified that the CCR2^+^ macrophages were dramatically reduced in AAA lesions from *Ninj1^ΔMΦ^* mice (**Figure** **6**A,B), and the expression level of CCR2 was positively correlated with the expression level of NINJ1 in Raw264.7 cells (Figure 6C; Figure S7A,B, Supporting Information). Overexpression of CCR2 increased the cell adhesion and trans‐endothelial migration of Raw264.7 cells (Figures 6D,E and S7C–E, Supporting Information). These results suggested that NINJ1 might modulate macrophage infiltration by regulating the expression level of CCR2.

NINJ1 was reported to activate the Toll‐like receptor 4 (TLR4) signaling in endothelial cells.^[^ ^26^ ^]^ It was confirmed that it also activated the TLR4 signaling in macrophages (Figure 6F‐J), demonstrating that overexpression of NINJ1 in Raw264.7 increased the expression levels of TLR4 and MyD88, and activated the phosphorylation of transcription factor NF‐κB p65 (Figure 6F–J). Consistently, the expression levels of TLR4, MyD88, and phosphor‐NF‐κB p65 were significantly decreased in the abdominal aortic tissues of *Ninj1^ΔMΦ^* mice compared with *Ninj1^fl/fl^* mice (Figure S8A–F, Supporting Information). TLR4 has been noted to play roles in macrophage infiltration, while the underlying mechanism has still remained elusive.^[^ ^36^ ^]^ To determine whether TLR4 signaling pathway could upregulate CCR2 expression level, Raw264.7 cells were treated with TLR4 inhibitor TAK‐242. After inhibition of TLR4, the expression level of CCR2 was reduced (Figure 6K). Furthermore, to examine whether NF‐κB p65 could regulate the transcription of *CCR2*, a dual‐luciferase reporter assay was carried out, and it was identified that overexpression of NF‐κB p65 has resulted in increased signals of luciferase linked with the *CCR2* promoter (Figure 6L), indicating that NF‐κB p65 has the potential to directly transcribe CCR2. Therefore, these findings strongly suggested that NINJ1 could facilitate macrophage infiltration through the TLR4/NF‐κB/CCR2 signaling pathway.

### 2.7 NINJ1 Activated TLR4 via Blocking the Interaction between TLR4 and Its Negative Regulator, ANXA2

**Figure 7:**
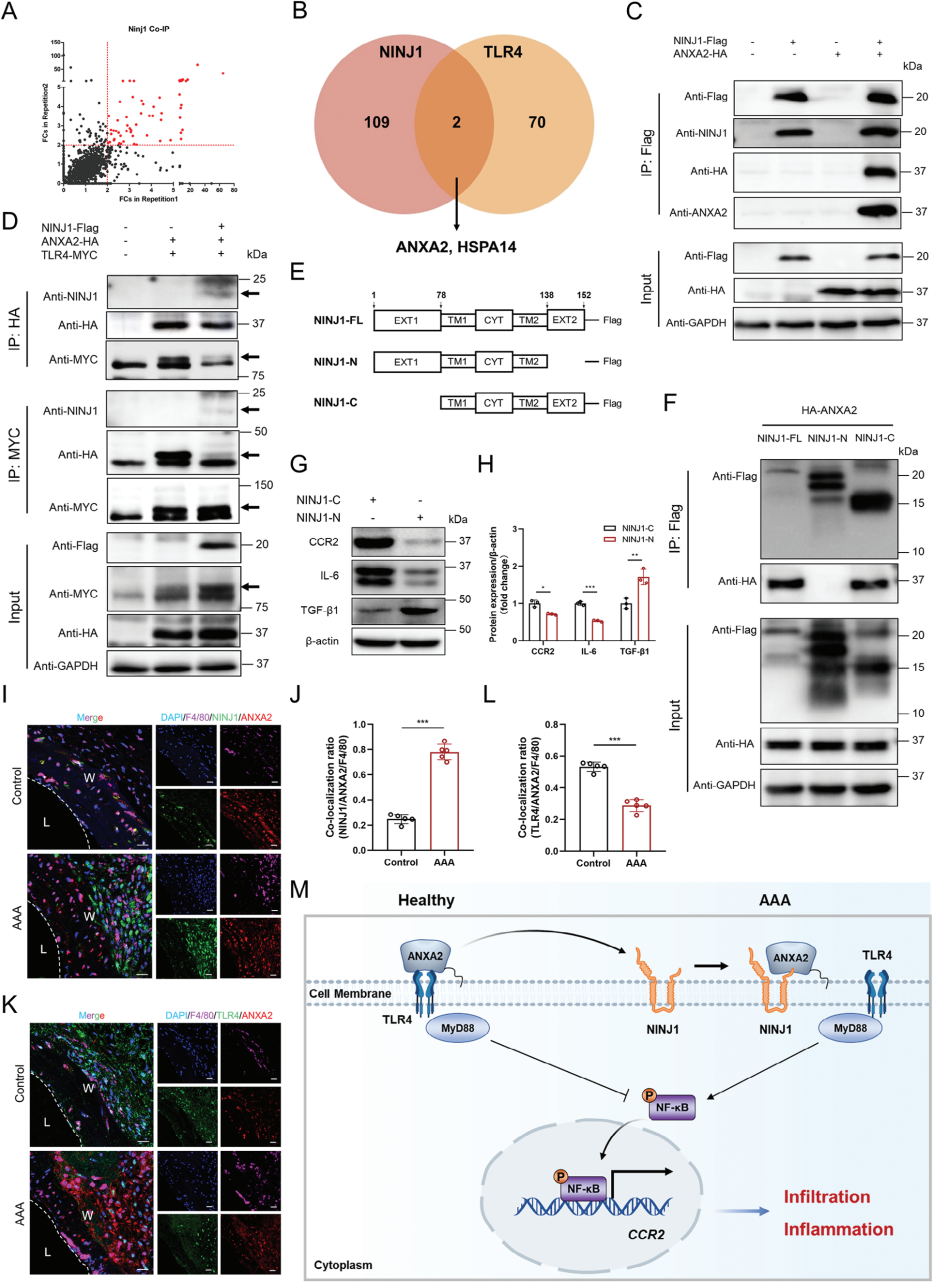
NINJ1 activated TLR4 by blocking the interaction between TLR4 and ANXA2. A) NINJ1‐interacted proteins identified by mass spectrometry. Proteins with a fold change (FC) > 2 were marked in red. B) Venn diagrams reveal NINJ1‐binding proteins identified by mass spectrometry and TLR4‐binding proteins identified from the Integrated Interactions Database. C) Verification of the interaction between NINJ1 and ANXA2. HEK293T cells were co‐transfected with Flag‐tagged NINJ1 and HA‐tagged ANXA2 plasmids. NINJ1 protein was immunoprecipitated by anti‐Flag affinity gel, and the protein expression levels of ANXA2 and HA in the precipitation were detected by Western blotting. D) Co‐immunoprecipitation (co‐IP) assay examining the interactions between NINJ1, ANXA2, and TLR4 proteins in HEK293T cells. ANXA2 or TLR4 was immunoprecipitated from cell lysates, and the presence of the indicated proteins in the precipitate was assessed via Western blotting. The arrows indicate target bands. E) Structures of wild‐type NINJ1 (full length, 1st‐‐152th amino acids) and its 2 truncations. F) Representative Western blotting images of co‐IP assay using anti‐Flag affinity gel in HEK293T cells. G) and H) Western blot analysis and quantification of CCR2, IL‐6, and TGF‐β1 expressions in Raw264.7 cells transfected with NINJ1‐N or NINJ1‐C plasmids. The level of β‐actin protein was used for normalization. Data were analyzed by the Student's *t*‐test; *n* = 3. ^*^ *p* < 0.05; ^**^ *p* < 0.01; ^***^ *p* < 0.001. I) and J) Immunofluorescence staining for NINJ1 (green) and ANXA2 (red) located with F4/80 (purple) in murine AAA and normal aortic tissues. Nuclei were stained by DAPI (blue). Scale bar = 20 µm. Data were analyzed by the Student's *t*‐test; *n* = 5. ^***^ *p* < 0.001. L, lumen; W, aortic wall. K) and L) Immunofluorescence staining for TLR4 (green) and ANXA2 (red) located with F4/80 (purple) in murine AAA and normal aortic tissues. Nuclei were stained by DAPI (blue). Scale bar = 20 µm. Data were analyzed by the Student's *t*‐test; *n* = 5. ^***^ *p* < 0.001. L, lumen; W, aortic wall. M) Schematic diagram of the NINJ1‐mediated pro‐inflammatory mechanism.

In order to assess the molecular mechanisms by which NINJ1 could activate TLR4, immunoprecipitation‐mass spectrometry (IP‐MS) was employed to screen the interactome of NINJ1. Through 2 independent repetitions, a total of 111 proteins were identified that interacted with NINJ1 (**Figure** **7**A; Table S5, Supporting Information), while TLR4 was not among them. This finding aligns with existing literature, indicating that NINJ1 does not have direct interactions with TLR4.^[^ ^26^ ^]^ within the pool of identified interacted proteins of NINJ1, 2 proteins, Annexin A2 (ANXA2) and heat shock 70 kDa protein 14 (HSPA14), were found that have been reported to bind with TLR4 (Figure 7B; Table S6, Supporting Information). Given the fact that both NINJ1 and TLR4 are membrane proteins, our concentration shifted toward ANXA2, a secreted and cell membrane‐located protein. The interaction between NINJ1 and ANXA2 was confirmed through co‐immunoprecipitation (co‐IP) and immunoblotting analysis (Figure 7C), as well as the interaction between TLR4 and ANXA2 (Figure S9A, Supporting Information). Furthermore, the subcellular colocalizations of NINJ1, ANXA2, and TLR4 were validated through co‐immunofluorescence staining (Figure S9B,C, Supporting Information). Notably, ANXA2 has been noted as a negative regulator of TLR4, binding to TLR4 and blocking its downstream signaling activation.^[^ ^37^ ^]^ To further determine the role of the NINJ1‐ANXA2 interaction in TLR4 signaling activation, all 3 proteins were co‐expressed and a pull‐down assay was carried out targeting ANXA2 and TLR4, respectively. A reduction was found in the interaction between ANXA2 and TLR4 when co‐expressed with NINJ1 compared with the NINJ1 null group (Figure 7D). This indicated that NINJ1 could competitively bind with ANXA2, preventing ANXA2 from binding to TLR4. To identify the specific domain of NINJ1 that directly bounds to ANXA2, HA‐tagged ANXA2 and 2 Flag‐tagged truncated forms of NINJ1, namely NINJ1‐N (1st–137th amino acids, lacking C‐termini), NINJ1‐C (79th–152th amino acids, lacking N‐termini), were constructed and introduced into HEK293T cells (Figure 7E). Co‐IP results demonstrated that only NINJ1 with full length and NINJ1‐C could interact with ANXA2, while NINJ1‐N failed in establishing interaction with ANXA2 (Figure 7F), indicating that the 138th–152th amino acids of NINJ1 were indispensable for the interaction between NINJ1 and ANXA2. Further studies were conducted to examine the impact of NINJ1‐ANXA2 interaction on macrophage inflammatory phenotype. NINJ1‐C or NINJ1‐N plasmids were expressed in macrophages, and the NINJ1‐N truncation reduced the expressions of pro‐inflammatory markers CCR2 and IL‐6, while rescuing the expression of anti‐inflammatory marker TGF‐β1 (Figure 7G,H). The immunofluorescence staining observed consistent results in normal and AAA tissues (Figure 7I–L). Collectively, these results suggested that NINJ1 could play a role in releasing and activating TLR4 by inhibiting the interaction between TLR4 and its negative regulator, ANXA2. This mechanism ultimately led to the increased expression level of CCR2 and enhanced macrophage infiltration (Figure 7M).

## 3 Discussion

The present study provided evidence that macrophage‐derived NINJ1 plays a crucial role in the development of AAA. The findings revealed a significant upregulation of NINJ1 in both human and murine AAA tissues, with a particular emphasis on its expression level in macrophages. Notably, it was observed that the deficiency of NINJ1 in macrophages led to a reduced incidence and severity of AAA in an Ang II‐induced murine model accompanied by decreased macrophage infiltration and mitigated macrophage‐mediated inflammation. Through comprehensive in vivo and in vitro analyses, we further elucidated the mechanisms underlying the contribution of NINJ1 to AAA pathogenesis. It was found that NINJ1 promoted a pro‐inflammatory macrophage phenotype and enhanced the adhesion and migratory capabilities. Intriguingly, it was unraveled that NINJ1 activated the TLR4/NF‐κB/CCR2 signaling pathway by competitively interacting with ANXA2. This activation ultimately facilitated macrophage infiltration and AAA formation. Hence, the present study highlighted the significance of macrophage‐derived NINJ1 as a key regulator in the development of AAA. By unraveling the molecular mechanisms associated with its effects on inflammation, adhesion, migration, and the TLR4/NF‐κB/CCR2 signaling pathway, valuable insights were provided into potential therapeutic targets for the management of AAA.

NINJ1 is a cell surface protein, which was first discovered and known as a core regulator of nerve repair and regeneration.^[^ ^38^ ^]^ In addition to its classical functions in controlling nerve regeneration, recent studies have found that NINJ1 participates in the pathogenesis of cancer, immune disease, and cardiovascular disease by regulating vascular homeostasis and inflammatory response.^[^ ^27^, ^32^, ^39^, ^40^ ^]^ Emerging evidence revealed that NINJ1 regulates cardiovascular physiology and pathophysiology.^[^ ^29^, ^30^, ^31^ ^]^ In the present study, it was indicated that NINJ1 was highly expressed in AAA lesions, and a detrimental influence of macrophage‐derived NINJ1 on AAA formation was identified. It is mainly consistent with previously reported findings, in which NINJ1 was predominantly expressed in myeloid cells at the inflammatory sites.^[^ ^18^, ^19^, ^24^ ^]^

Macrophage infiltration plays a critical role in the pathogenesis of AAA, contributing to the production of pro‐inflammatory cytokines, proteolytic enzymes, and oxidation‐derived free radicals, which exacerbate inflammation and aortic wall damage.^[^ ^13^ ^]^ In this study, we observed that macrophage NINJ1 deficiency suppressed macrophage infiltration in the aortic wall of a murine AAA model. Additionally, in vitro experiments showed that NINJ1 promoted the adhesion and trans‐endothelial migration ability of macrophages. These findings align with previous research on inflammation in the central nervous system, which demonstrated that global *Ninj1* knockout or NINJ1‐blocking antibody inhibited the transmigration of pro‐inflammatory myeloid cells across the blood‐brain barrier.^[^ ^19^, ^23^ ^]^ Ahn et al reported that NINJ1 overexpression enhanced basal motility and trans‐endothelial migration of Raw264.7 cells by promoting filopodial projection formation.^[^ ^41^ ^]^ Notably, it has been reported that NINJ1 does not affect macrophage infiltration in experimental colitis lesions,^[^ ^18^ ^]^ and inhibits macrophage inflammation in atherosclerosis.^[^ ^29^ ^]^ These observations suggest that the biological functions of NINJ1 may exhibit tissue and disease specificity. The soluble form of NINJ1 (sNINJ1) liberated by MMP9 could act as a chemoattractant for macrophages.^[^ ^42^ ^]^ However, Jeon et al demonstrated that sNINJ1 inhibited monocyte recruitment and macrophage accumulation in the context of atherosclerosis.^[^ ^29^ ^]^ As the expression of NINJ1 and MMP9 increased in AAA tissues, we observed increased expression of sNINJ1 in human serum samples, but not in mice, and the proportional increase of sNINJ1 was not as significant as that of NINJ1 in human samples, suggesting that the potential anti‐inflammatory effects of sNINJ1 may not counteract the pro‐inflammatory effects of NINJ1 in AAA pathogenesis overall.

CCR2 is a well‐established regulator that plays a noticeable role in facilitating the migration of pro‐inflammatory monocytes/macrophages from the circulation to sites of vascular inflammation.^[^ ^35^ ^]^ In a study conducted by Moran et al, it was demonstrated that the infiltration of CCR2‐positive monocytes in the aorta exhibited a positive correlation with the maximum diameter of the aorta in a murine AAA model.^[^ ^43^ ^]^ Additionally, both global and leukocyte‐specific knockout of *Ccr2* led to significant inhibition of murine AAA formation, which was attributed to a substantial reduction in macrophage‐mediated inflammation.^[^ ^44^, ^45^ ^]^ Our findings further support the involvement of NINJ1 in regulating CCR2 expression in macrophages. We observed that NINJ1 promoted CCR2 expression level by upregulating the phosphorylation of NF‐κB p65. Moreover, the mechanistic experiments revealed that NF‐κB p65 directly facilitated the transcription of the CCR2 gene. Collectively, these data suggest that CCR2 may represent one of the primary targets through which NINJ1 influences the development of AAA.

It is widely acknowledged that TLR4 plays a remarkable role in regulating inflammation and injury in cardiovascular diseases.^[^ ^46^ ^]^ Studies have shown that TLR4 expression level is elevated in human AAA, and it is associated with an increased risk of large AAA.^[^ ^47^ ^]^ Additionally, blocking TLR4 and its downstream signaling pathway has been found to protect against AAA development by inhibiting the inflammatory process.^[^ ^48^ ^]^ Jennewein et al demonstrated that NINJ1 contributes to LPS‐triggered systemic inflammation by mediating TLR4 signaling pathway through activator protein‐1 activation and p38 phosphorylation in arterial endothelial cells.^[^ ^26^ ^]^ However, the specific molecular mechanisms underlying the regulation of TLR4 signaling pathway by NINJ1 have not yet been fully explored. In the present study, we provided evidence suggesting that NINJ1 activates TLR4 by competitively binding with ANXA2, thereby blocking the interaction between TLR4 and ANXA2. ANXA2, a negative regulator of TLR4, binds to TLR4 and inhibits its downstream signaling activation.^[^ ^37^ ^]^ Notably, we identified 2 proteins, ANXA2 and HSPA14, that interact with both NINJ1 and TLR4. HSPA14 is a component of the ribosome‐associated complex and acts as a potent immunoadjuvant produced under stress conditions, but not under physiological conditions.^[^ ^49^ ^]^ These findings uncover a novel molecular mechanism through which NINJ1 activates the TLR4 signaling pathway.

Previous studies have shed light on the involvement of various genes in the initiation and progression of AAA. For instance, Gasdermin D (*GSDMD*),^[^ ^50^ ^]^ *NR1D1*,^[^ ^51^ ^]^ and *TFEB* ^[^ ^52^ ^]^ derived from VSMCs, have been identified as key regulators of AAA development by modulating VSMC apoptosis, mitochondria metabolism, and phenotype switching. On the other hand, genes, such as *ADAR1*,^[^ ^53^ ^]^ *JMJD3*,^[^ ^54^ ^]^ and *BAM1*,^[^ ^55^ ^]^ derived from macrophages, contribute to vascular inflammation and aneurysm formation. In the present study, we discovered that NINJ1, derived from macrophages, plays a crucial role in AAA development by promoting macrophage activation and infiltration. It is worth noting that other cell types, including endothelial cells, fibroblasts, and hematopoietic stem cells, are also involved in AAA pathogenesis through distinct mechanisms.^[^ ^56^, ^57^, ^58^ ^]^ Gasdermin D, known for its role in pyroptosis, has been demonstrated to contribute to the development of AAA.^[^ ^50^ ^]^ Notably, NINJ1 has recently been implicated in various forms of lytic cell death, including pyroptosis, apoptosis, and necrosis,^[^ ^27^ ^]^ and we also found reduced expression of lytic cell death markers in aortic tissue of NINJ1‐deficient mice, suggesting its potential versatility in regulating AAA.

There were several limitations that were acknowledged in our study. First, the clinical AAA tissues used in this study were obtained from patients who required open surgical procedures, which may have introduced a selection bias and limited our insights into the early stages of AAA development. Obtaining tissues from patients at earlier disease stages would have provided a more comprehensive understanding of AAA initiation. Second, it is important to note that there was no available animal model at the time that fully recapitulated the complex pathophysiology of AAA observed in human patients. In this study, we employed the classic Ang II infusion method to induce AAA formation in mice, which replicated key pathological features of human AAA.^[^ ^59^ ^]^ However, validation of our findings in other AAA models would have enhanced the robustness and generalizability of our results. Lastly, although we demonstrated that macrophage NINJ1 deficiency attenuated AAA formation, further studies were needed to evaluate the therapeutic potential of targeting NINJ1 in vivo. Conducting therapeutic interventions targeting NINJ1 in animal models of AAA would have provided valuable insights into its specific role as a potential therapeutic target for AAA. Addressing these limitations and further investigating the role of NINJ1 in early‐stage AAA development and therapeutic interventions may enhance our understanding of AAA pathogenesis and facilitate the identification of novel strategies for the treatment of this challenging disease.

In conclusion, the present study provided novel insights into the role of NINJ1 in AAA pathogenesis. It was identified that high expression of NINJ1 was observed in AAA lesions, specifically in macrophages. Strong evidence was provided indicating that AAA formation was facilitated by macrophage‐derived NINJ1 through the promotion of macrophage infiltration into the aortic walls. Additionally, it was discovered that NINJ1 activated the TLR4/NF‐κB/CCR2 pathway by blocking the interaction between TLR4 and ANXA2. This finding emphasizes the potential of NINJ1 as a therapeutic target for the prevention or treatment of AAA.

## 4 Experimental Section

### Patients and Specimens

This study was performed in accordance with the principles of the Declaration of Helsinki. All participants provided informed consent prior to enrollment and the study protocol was approved by the Ethics Committee of Shanghai Ninth People's Hospital, Shanghai JiaoTong University School of Medicine (Shanghai, China; Approval No.: SH9H‐2021‐TK32‐1). The analysis of human samples and retrospective clinical data was conducted in accordance with the guidelines and regulations approved by the Institutional Review Board of Shanghai Jiao Tong University. Human AAA tissue samples were collected from 3 patients who underwent open surgery for AAA between September 2019 and May 2020. Additionally, aortic tissues from 3 organ‐transplant donors were obtained and utilized as controls for making comparisons. AAA was defined as dilation of the abdominal aorta to ≥3.0 cm in diameter in accordance with the Practice Guidelines released by the American Heart Association/American College of Cardiology Joint Committee.^[^ ^3^ ^]^ The aortic tissues were fixed with 4% paraformaldehyde and used for subsequent immunofluorescence assay. Serum samples were collected from a total of 14 AAA patients and 10 control subjects (patients with deep venous thrombosis) between January 2021 and March 2022 (Table S1, Supporting Information). The blood samples were obtained from all subjects in a fasting state between 7 am and 8 am. Following collection, the serum samples were stored at −80 °C for subsequent analysis. The concentrations of NINJ1 in serum from patients were detected using an enzyme‐linked immunosorbent assay (ELISA) kit (Cusabio, China).

### Animals

The animal experiments were approved by the Animal Ethics Committee of Shanghai Ninth People's Hospital, Shanghai JiaoTong University School of Medicine (Approval No.: SH9H‐2021‐A087‐SB). Myeloid conditional *Ninj1* knockout mice (*Ninj1^flox/flox^Lyz2‐Cre*) were generated by breeding *Ninj1^flox/flox^* mice from Shanghai Model Organisms Center (Shanghai, China) with *Lyz2‐Cre* [B6.129P2‐*Lyz2*1/J, #0 04781] mice from Jackson Laboratory (Farmington, CT, USA). Subsequently, *Ninj1^flox/flox^Lyz2‐Cre* mice were crossed with *ApoE^−/−^* mice to establish *ApoE^−/−^Ninj1^flox/flox^Lyz2‐Cre* (abbreviated as *Ninj1^ΔMΦ^*) mice. *ApoE^−/−^Ninj1^flox/flox^* (abbreviated as *Ninj1^fl/fl^*) littermates were utilized as controls. All mice were kept in a clean room with free access to water and food under a 12‐h light/dark cycle.

### Establishment and Evaluation of the Murine AAA Model

The angiotensin II (Ang II)‐induced murine AAA model was established as previously described.^[^ ^60^ ^]^ Briefly, 12‐ to 16‐week‐old male mice were anesthetized by isoflurane inhalation (RWD Life Science Co., Ltd., Shenzhen, China; induction dose: 3%–5%, maintenance dose: 1%–2%). Then, mini osmotic pumps (Alzet model 2004; Durect Corporation, Cupertino, CA, USA) loaded with 1000 ng min^−1^ kg^−1^ of Ang II (A9525; Sigma‐Aldrich, St. Louis, MO, USA) or saline (0.9% NaCl) were implanted subcutaneously at the back of the neck for 28 days. The systolic blood pressure (SBP) of mice was determined using a noninvasive tail‐cuff system (BP‐2000 Blood Pressure Analysis System, Visitech Systems, New York, NY, USA). To detect the formation of AAA in vivo, micro‐ultrasound imaging was carried out using a Vevo770 High‐Resolution In vivo Micro‐Imaging system (VisualSonics, Toronto, Canada). At 28 days after implantation surgery, mice were humanely euthanized with an inhalation overdose (5%) of isoflurane and cervical dislocation, and the suprarenal abdominal aortas and spleens were harvested for further analysis. An autopsy was performed on mice that had died prior to reaching the study endpoint. The severity of AAA was classified into 4 types: type I, an enlargement that was 1.5–2 times the diameter of a normal abdominal aorta; type II, a single dilation that was more than 2 times the diameter of a normal abdominal aorta; type III, multiple dilations in the suprarenal region; type IV, death due to aneurysmal rupture, characterized by the presence of crevasse in the dilated aortic wall with blood clots in the retroperitoneum.^[^ ^61^ ^]^ Blood samples from mice were centrifuged at 3500 rpm for 10 min at 4 °C, and serum was collected for determination of the levels of NINJ1, interleukin‐1β (IL‐1β), tumor necrosis factor‐α (TNF‐α), IL‐6, C‐C motif chemokine ligand 2 (CCL2), transforming growth factor‐β1 (TGF‐β1) and IL‐10 using commercial ELISA kits (Cusabio, China). All measurements and analyses were conducted by 2 trained, independent observers who were blinded to the genotype and treatment conditions.

### Statistical Analysis

Statistical analysis was conducted using GraphPad Prism 8.0 software (GraphPad Software Inc., San Diego, CA, USA). Categorical variables were presented as the number (percentage), and continuous data were expressed as mean ± standard error of the mean (SEM). The normality of data distribution was assessed using the Shapiro‐Wilk test. For normally distributed variables, unpaired two‐tailed Student's *t*‐test was applied to determine statistically significant differences between 2 groups, and one‐way analysis of variance (ANOVA) or two‐way ANOVA, followed by Bonferroni post hoc analysis was used for making multiple comparisons. For abnormally distributed variables, the Mann‐Whitney U test was utilized. A comparison of bivariate categorical variables was conducted using the χ^2^ test or Fisher's exact test. Survival curves were evaluated by the Kaplan‐Meier method combined with the log‐rank test. *p* < 0.05 was considered statistically significant.

## Conflict of Interest

The authors declare no conflict of interest.

## Author Contributions

Z.W., Z.X., and H.P. contributed equally to this work. Z.X., B.L., X.W., and X.L. designed and supervised the experiments, and revised the manuscript. Z. Wu, Z. Xu, and H. Pu wrote the manuscript and analyzed the data. Z.W., Z.X., H.P., A.D., J.H., J.L., C.Z., P.Q., J.Q., X.W., and B.L. performed experiments. X.W. and X.L. collected and assessed clinical samples. The order of the co‐first authors was determined based on their contributions to the manuscript. All authors have reviewed and approved the final version of the manuscript.

## Supporting information

Supporting Information

## Data Availability

The data that support the findings of this study are available from the corresponding author upon reasonable request.
