# Supplementary material for: NINJ1 Facilitates Abdominal Aortic Aneurysm Formation via Blocking TLR4‐ANXA2 Interaction and Enhancing Macrophage Infiltration
Source: Adv Sci (Weinh). 2024 Jun 23;11(31):2306237. doi: 10.1002/advs.202306237 (PMC11336960; doi:10.1002/advs.202306237)
Supplement: Supplementary file 1 — Supporting Information [file ADVS-11-2306237-s001.docx]

Supporting information

for

**NINJ1 Facilitates Abdominal Aortic Aneurysm Formation via Blocking TLR4-ANXA2 Interaction and Enhancing Macrophage Infiltration**

Zhaoyu Wu, Zhijue Xu*, Hongji Pu, Ang’ang Ding, Jiateng Hu, Jiahao Lei, Chenlin Zeng, Peng Qiu, Jinbao Qin, Xiaoyu Wu, Bo Li*, Xin Wang*, Xinwu Lu*

Z. Wu, Z. Xu, H. Pu, J. Hu, J. Lei, C. Zeng, P. Qiu, J. Qin, X. Wu, B. Li, X. Wang, X. Lu

Department of Vascular Surgery

Shanghai Ninth People’s Hospital, Shanghai JiaoTong University School of Medicine

Shanghai 200011, China

Z. Xu

Key Laboratory of Systems Biomedicine (Ministry of Education), Shanghai Center for Systems Biomedicine, Shanghai Jiao Tong University

Shanghai 200240, China

Z. Wu, P. Qiu, J. Qin, X. Wu, X. Wang, X. Lu

Vascular Center of Shanghai JiaoTong University

Shanghai 200011, China

A. Ding

Department of Ultrasound

Shanghai Ninth People’s Hospital, Shanghai JiaoTong University School of Medicine

Shanghai 200011, China

* Corresponding author. E-mail: boli@shsmu.edu.cn (B. Li); xuzhijue@sjtu.edu.cn (Z. Xu); wangxin9h@shsmu.edu.cn (X. Wang); luxinwu@shsmu.edu.cn (X. Lu)

**Supplementary Methods**

***Bioinformatics Analysis:*** The RNA array dataset of GSE7084 from the Gene Expression Omnibus (GEO) database (https://www.ncbi.nlm.nih.gov/geo/query/acc.cgi?acc=GSE7084) was utilized to analyze the expression level of *NINJ1* in human normal and AAA tissues. Gene Ontology (GO) enrichment analysis was performed using R 4.2.1 software (http://www.r-project.org/).

***Histopathological and Immunohistochemical Analyses:*** The aorta samples from human and mice were fixed with 4% paraformaldehyde, and were then embedded into paraffin to prepare sections. Hematoxylin and eosin (HE), Masson's trichrome, Elastica-van Gieson (EVG) or terminal deoxyuridine 5′-triphosphate nick-end-labelling (TUNEL) staining was conducted on the deparaffinized sections. The content of collagen and elastin degradation in the aortic wall were quantified. As previously described,^[1]^ elastin degradation was categorized into four categories: grade 1, no degradation in elastic laminae; grade 2, some interruptions; grade 3, multiple interruptions; grade 4, extreme fragmentation or loss. For immunohistochemical analysis, deparaffinized murine aorta sections were blocked with phosphate-buffered saline (PBS) containing 3% bovine serum albumin (BSA; 4240GR100, Biofroxx, Berlin, German), followed by overnight incubation with primary antibodies targeting MMP2, MMP3, MMP9, cleaved caspase-1 (c-CASP1), and high mobility group box 1 (HMGB1) at 4 °C. The details of the primary antibodies are listed in Table S2. Subsequently, the sections were incubated with appropriate secondary antibodies at room temperature for 1 h. All sections were visualized using an upright microscope (BX-51, Olympus, Tokyo, Japan) according to the manufacturer’s instructions. Quantification analysis was carried out on the average of five selected fields using ImageJ software (National Institutes of Health, Bethesda, MD, USA). To minimize technical variations, all samples were stained and analyzed simultaneously for each experiment.

***Immunofluorescence Analysis:*** Immunostaining for NINJ1, F4/80, CD31, α-SMA, INOS, CD206, CCR2, TLR4, MyD88, phospho-NF-κB p65, and ANXA2 was performed following the protocol described below. Deparaffinized sections were rinsed with PBS and permeabilized using 0.01% Triton X-100. Sequential co-immunofluorescence staining was conducted as previously outlined.^[2]^ Briefly, sections were incubated with PBS containing 3% BSA and then appropriately diluted with primary antibodies at 4 °C overnight. The details of primary antibodies are listed in Table S2. Subsequently, the sections were incubated with a suitable combination of corresponding Alexa Fluor 488-, 594-, and 647-conjugated secondary antibodies (Invitrogen, Carlsbad, CA, USA) at room temperature for 1 h. Finally, sections were stained with 4′,6-diamidino-2-phenylindole (DAPI) and observed under a fluorescence microscope (Olympus).

***Single-Cell RNA-Sequencing (scRNA-seq):*** Murine aortic tissues were harvested, minced, and digested in 0.2% Collagenase II (Life Technologies) and 0.2% Collagenase V (Sigma-Aldrich) in plain medium at 37°C for 1 h, and then strained through a 70 μM mesh. The samples were sequenced at the University of Michigan Advanced Genomics Core on the 10X Chromium system on an Illumina NovaSeq 6000 platform. Demultiplexing and gene quantification was carried out using the Cell Ranger software (version 7.0.0, 10x Genomics). Data analysis and visualization were conducted using the Loupe Browser software (version 6.5.0, 10x Genomics).

***Isolation of BMDMs:*** Bone-marrow derived macrophages (BMDMs) were isolated as previously described.^[3]^ Femur and tibia were collected from 8-week-old mice and placed into Petri dishes containing serum-free Dulbecco’s Modified Eagle’s medium (DMEM; Gibco, New York, NY, USA) with small cuts at each end. Marrow was flushed out into cold PBS containing 2% FBS using needles. The bone marrow was then incubated with red blood cell lysis buffer at room temperature for 10 min and subsequently centrifuged at 500 g for 5 min. The cell pellet was resuspended and aliquoted in 10 cm Petri dishes containing RPMI-1640 medium (11875093; Gibco) supplemented with 20 ng mL^-1^ macrophage colony-stimulating factor (M-CSF; 315-02; PeproTech, Cranbury, NJ, USA), 1% penicillin-streptomycin (15140-122; Gibco), and 10% FBS (10099141; Gibco). M-CSF treatment was performed every 48 h for 7 days to induce monocyte differentiation into macrophages. On day 7, total RNA from BMDMs was collected for RT-qPCR and RNA-sequencing (RNA-seq) analyses.

***Bulk RNA-Seq and Data Analysis:*** The RNA libraries were established utilizing NEBNext Ultra RNA Library Prep kit for Illumina (New England Biolabs, USA), and they were then submitted for whole mRNA-seq on the Illumina NovaSeq platform. The mouse reference genome was used as the alignment target for the whole RNA next-generation sequencing reads, employing the STAR aligner (<http://code.google.com/p/rna-star/>). HTseq, a Python package, was utilized to generate gene-level read summarization. The analysis of differentially expressed genes (DEGs) was carried out using the DESeq2 R package. Principal component analysis (PCA) of the RNA-seq data was conducted using the factoextra R package. The gene expression intensity was visualized using Multiple Experimental Viewer (MeV) 4.9.0 software (http://www.tm4.org/mev/). For GO, Reactome, and Kyoto Encyclopedia of Genes and Genomes (KEGG) pathway enrichment analyses, the clusterProfiler R package and Gene Set Enrichment Analysis (GSEA) 4.1.0 software (<http://www.broadinstitute.org/gsea>) were utilized.

***Cell Culture and Transfection:*** Raw264.7 macrophage (RRID: CVCL_0493) and HEK293T (RRID: CVCL_0063) cell lines were purchased from the American Type Culture Collection (USA) and cultivated in a DMEM (11995500, Gibco) supplemented with 10% FBS and 1% penicillin-streptomycin in a 5% CO_2_ incubator at 37 °C. Human umbilical vein endothelial cells (HUVECs; RRID: CVCL_2959) were purchased from ScienCell Research Laboratories (Carlsbad, CA, USA) and cultured in an endothelial cell medium (ECM, ScienCell Research Laboratories). A stock solution of TAK-242 (CLI-095, MCE, USA) at a concentration of 10 mM was prepared in dimethylsulfoxide (DMSO) and stored at -20 °C. To construct the eukaryotic expression vector of target gene or siRNA, the PCR-amplified fragments were inserted into pcDNA3.1 (Invitrogen). The shRNA and siRNA sequences are listed in Table S3. The lentiviral vector expressing *Ninj1* shRNA was constructed by introducing the *Ninj1* shRNA fragment into the pmiRZip vector (System Biosciences, Palo Alto, CA, USA). HEK293T cells were co-transfected with recombinant lentivirus vector and pMD2.G Packaging Plasmid Mix (12259, Addgene) using Advanced Transfection reagent (AD600075, ZETA LIFE, CA, USA) according to the manufacturer’s instructions. Then, the lentivirus was harvested to infect Raw264.7 cells. Transfected cell lines were selected using puromycin. Furthermore, siRNAs or plasmid transfection was performed using the Advanced Transfection reagent (AD600075, ZETA LIFE) according to the manufacturer's instructions. After 24 h of transfection, the concentrations of IL-1β, TNF-α, IL-6, and CCL2 in the supernatants of Raw264.7 cells were determined using ELISA kits (Multiscience, Shanghai, China) following the manufacturer's instructions. In another set of experiments, Raw264.7 cells were transfected with wild-type NINJ1 and its truncations missing the 1st-78th amino acids (79th-152th amino acids, lacking N-termini) or 138th-152th amino acids (1st-137th amino acids, lacking C-termini) with the Advanced Transfection reagent (AD600075, ZETA LIFE) for 24 h.

***Macrophage Adhesion and Migration*** ***Assay:*** For macrophage and endothelial cell adhesion assay, 5×10^4^ HUVECs were seeded into a 12-well plate for 24 h. Raw264.7 cells, prelabeled with BCECF-AM (HY-101883, MCE, USA), were then seeded into the corresponding wells containing confluent monolayer of HUVECs for 30 min at 37 °C. Nonadherent cells were rinsed, and the number of adherent cells was counted under an Eclipse TE300 inverted microscope (Nikon, Tokyo, Japan). For macrophage trans-endothelial migration assay, 1.5×10^4^ HUVECs were seeded into the upper chamber of transwell inserts with 5.0 μm pores (Corning, NY, USA). Raw264.7 cells were suspended in a DMEM and added to the upper chamber at a density of 5×10^5^ cells per well. Afterwards, DMEM containing 10% FBS and 100 ng mL^-1^ of CCL2 (abs04173; Absin Bioscience Inc., Shanghai, China) was added to the lower chamber. After 24 h of incubation, cells that were migrated to the lower chambers were stained with crystal violet (C3886, Sigma-Aldrich) and subsequently observed using an inverted microscope (Nikon). Quantification analysis was performed on five selected fields using ImageJ software.

***Protein Extraction and Western Blot Assay:*** Total protein was extracted using 1% Triton X-100 (Sigma-Aldrich). The protein concentration was measured via a bicinchoninic acid (BCA) Protein Assay kit (23227; Thermo Fisher Scientific, Waltham, MA, USA). Proteins were separated by sodium dodecyl sulfate-polyacrylamide-gel electrophoresis (SDS-PAGE) and were then transferred onto polyvinylidene fluoride (PVDF) membranes (Beyotime, Shanghai, China). The protein bands were detected using a Tanon- 410 automatic gel imaging system (Tianneng, Huzhou, China). The primary antibodies are listed in Table S2.

***Total RNA Isolation and RT-qPCR Assay:*** Total RNA was isolated using standard TRIzol reagent (15596026, Invitrogen), and it was reversely transcribed using a First Strand cDNA Synthesis kit (G3330-100; Servicebio, Wuhan, China). The quantitative reverse transcription polymerase chain reaction (RT-qPCR) was performed on an ABI 7900HT RealTime PCR system (Life Technologies, Waltham, MA, USA) using the SYBR Green qPCR Master Mix (G3320-01, Servicebio). The ΔΔCt method was employed to calculate relative gene expression, and glyceraldehyde-3-phosphate dehydrogenase (*Gapdh*) was used as the reference gene. The gene-specific primer sequences for RT-qPCR are summarized in Table S4.

***Luciferase Reporter*** ***Assay:*** Luciferase reporter assay was carried out as previously described.^[4]^ HEK293T cells were transfected with the psiCHECK-2 luciferase reporter vector (Promega, Madison, WI, USA) containing human *CCR2* gene promotor, along with either the NF-κB p65-coding vector or blank pcDNA3.1 vector, using the Advanced Transfection reagent (AD600075, ZETA LIFE). After 48 h of transfection, cells were lysed with reporter lysis buffer, and luciferase activities were measured using a dual-luciferase reporter assay system (E1910, Promega). The relative luciferase activity was determined by calculating the ratio of luciferase units to Renilla units.

***Immunoprecipitation-Mass Spectrometry (IP-MS):*** For immunoprecipitation analysis of NINJ1 binding protein, HEK293T cells were infected with Flag-tagged NINJ1 plasmids (20585; Sangon Biotechnology Co., Ltd., Shanghai, China). Cell lysates were purified with anti-Flag affinity beads (36403, Yeasen Biotechnology Co., Ltd., Shanghai, China), and centrifuged at 1000 g for 10 min at 4 °C. The precipitates were incubated with 1 M dithiothreitol (DTT) for 1 h at 37 °C, and they were then incubated with iodoacetamide for 40 min in darkness. Subsequently, the precipitates were washed with 100 mM NH_4_HCO_3_ and treated with 1 µg trypsin and 100 mM CaCl_2_ at 37 °C overnight. Finally, isolated proteins were dried by vacuum centrifugation. The samples were examined using an Orbitrap Exploris 480 mass spectrometry system (Thermo Fisher Scientific) and analyzed by Proteome Discoverer 2.1 software.

***Co-Immunoprecipitation (Co-IP):*** HEK293T cells were co-transfected with Flag-tagged NINJ1 and HA-tagged ANXA2 plasmids or MYC-tagged TLR4 and HA-tagged ANXA2 plasmids. In another set of experiments, HEK293T cells were co-transfected with Flag-tagged NINJ1 truncations and HA-tagged ANXA2 plasmids. The total protein of cells was extracted, and 500 µg of proteins was incubated overnight at 4 °C with the indicated antibody. On the following day, 20 µl of protein A/G beads (36403, Yeasen) was added to the protein-antibody complex and incubated for 4 h at 4 °C. After centrifugation at 1000 g for 10 min at 4 °C, the precipitates were washed and then boiled in SDS buffer for 5 min. The protein expressions were analyzed by Western blotting.

***Immunofluorescence Co-Localization Analysis:*** HEK293T cells were seeded into glass bottom cell culture dish (801001; Nest Biotechnology, Woodbridge, USA), and they were then co-transfected with Flag-tagged NINJ1 or MYC-tagged TLR4 and HA-tagged ANXA2 plasmids for 24 h. Subsequently, the cells were fixed with 4% paraformaldehyde for 15 min, and they were further incubated with mouse anti-NINJ1 or mouse anti-TLR4 and rabbit anti-AXNA2 antibodies at 4 °C overnight. After washing, the cells were incubated with corresponding Alexa Fluor 594- and 488‐conjugated secondary antibodies at 37 °C for 2 h in the dark. Finally, the cells were incubated with DAPI at room temperature for 3 min. The fluorescence signals were visualized using an inverted fluorescence microscope (Nikon).

**References**

[1] Sun J, Sukhova GK, Yang M, Wolters PJ, MacFarlane LA, Libby P, Sun C, Zhang Y, Liu J, Ennis TL, Knispel R, Xiong W, Thompson RW, Baxter BT, Shi GP, *J Clin Invest* **2007**, *117*, 3359-3368.

[2] Sun LY, Lyu YY, Zhang HY, Shen Z, Lin GQ, Geng N, Wang YL, Huang L, Feng ZH, Guo X, Lin N, Ding S, Yuan AC, Zhang L, Qian K, Pu J, *Circulation* **2022**, *146*, 1591-1609.

[3] Ying W, Cheruku PS, Bazer FW, Safe SH,Zhou B, *J Vis Exp* **2013**, *76*, 50323.

[4] Liu Y, Li M, Lv X, Bao K, Yu Tian X, He L, Shi L, Zhu Y, Ai D, *Circ Res* **2022**, *130*, 851-867.

**Supplementary Figures and Figure Legends**


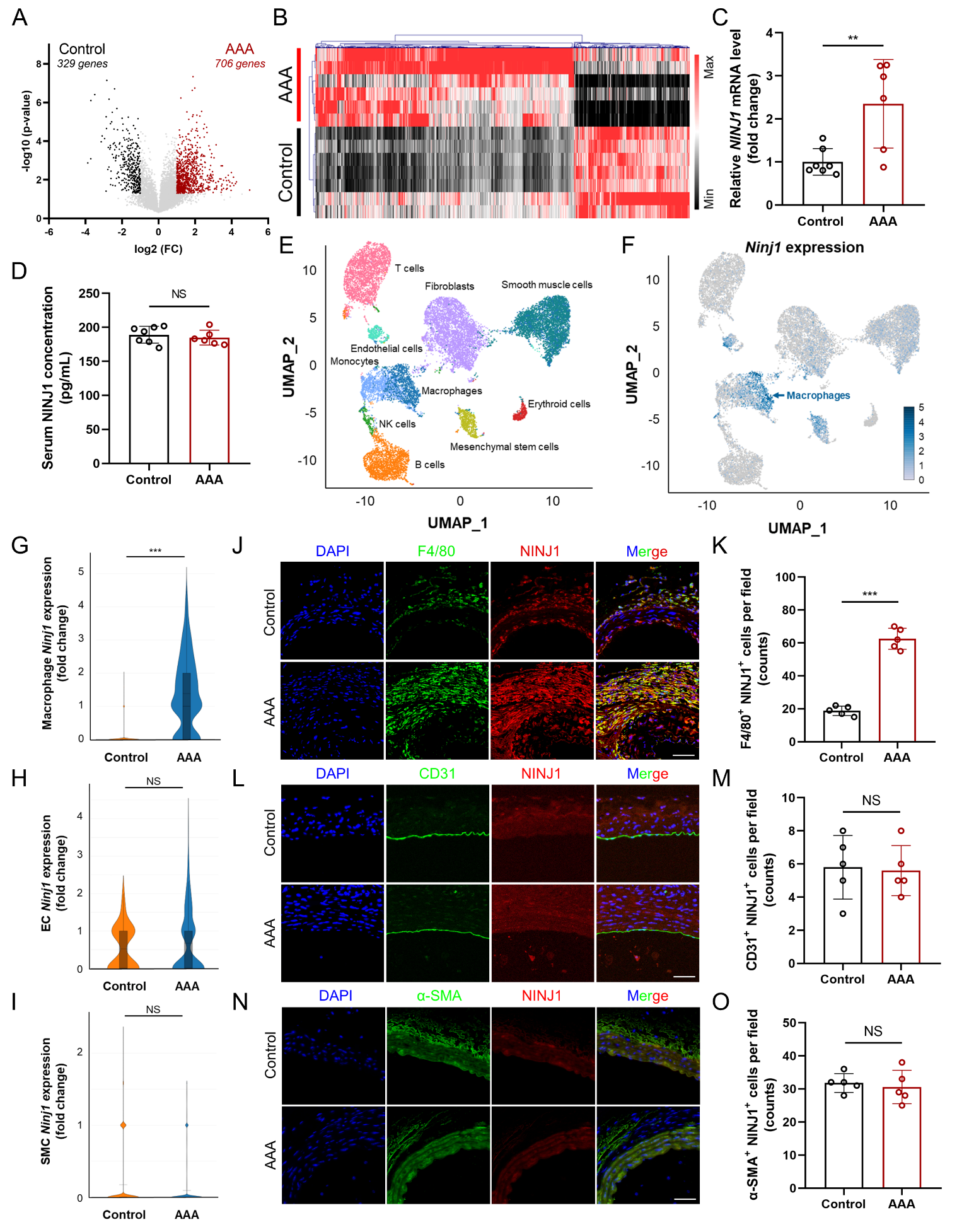


**Figure S1. Nerve injury-induced protein 1 (NINJ1) is elevated in macrophages of human and murine abdominal aortic aneurysm (AAA) tissues. (A)** Volcano plot of differentially expressed genes (DEGs) between human AAA (n = 6) and non-AAA (n = 7) tissues from GSE7084 dataset. DEGs were defined as genes with a |log2(fold-change)| ≥ 1 and *P* < 0.05. Upregulated genes were displayed in red, and downregulated genes were shown in black. **(B)** Heatmap of DEGs between human AAA and non-AAA tissues from GSE7084 dataset. Downregulated genes were shown in black, and upregulated genes were displayed in red. **(C)** Quantification of *NINJ1* RNA expression level in human AAA and non-AAA tissues from GSE7084 dataset. Data were analyzed by the Student’s *t*-test. ***P* < 0.01. **(D)** Serum samples from control (n = 7) and angiotensin II (Ang II)-induced AAA mice (n = 6) were analyzed using ELISA to measure NINJ1 protein concentration. Statistical analysis was performed using the Student’s *t*-test. NS indicates *P* > 0.05. **(E)** Cluster analysis using the uniform manifold approximation and projection (UMAP) technique of single cell RNA-sequencing from murine AAA (n = 5) and normal aortic (n = 5) samples. **(F)** Feature plots displaying the single-cell gene expression of *Ninj1* across cell clusters. Violin plots displaying Z score transformed expression levels of *Ninj1* in **(G)** macrophages, **(H)** endothelial cells (EC), and **(I)** smooth muscle cells (SMC). **(J)** and **(K)** Representative images of NINJ1 (red) expression level by immunofluorescence staining of murine AAA and non-AAA tissues, and co-staining with the key macrophage–associated marker F4/80 (green) and DAPI (blue). Scale bar = 50 μm. Data were analyzed by the Student’s *t*-test; n = 5. ****P* < 0.001. **(L)** and **(M)** Representative images of NINJ1 (red) expression level by immunofluorescence staining of murine AAA and non-AAA tissues, and co-staining with the endothelial cell–associated marker CD31 (green) and DAPI (blue). Scale bar = 50 μm. Data were analyzed by the Student’s *t*-test; n = 5. NS indicates *P* > 0.05. **(N)** and **(O)** Representative images of NINJ1 (red) expression level by immunofluorescence staining of murine AAA and non-AAA tissues, and co-staining with the vascular smooth muscle cells–associated marker α-SMA (green) and DAPI (blue). Scale bar = 50 μm. Data were analyzed by the Student’s *t*-test; n = 5. NS indicates *P* > 0.05.


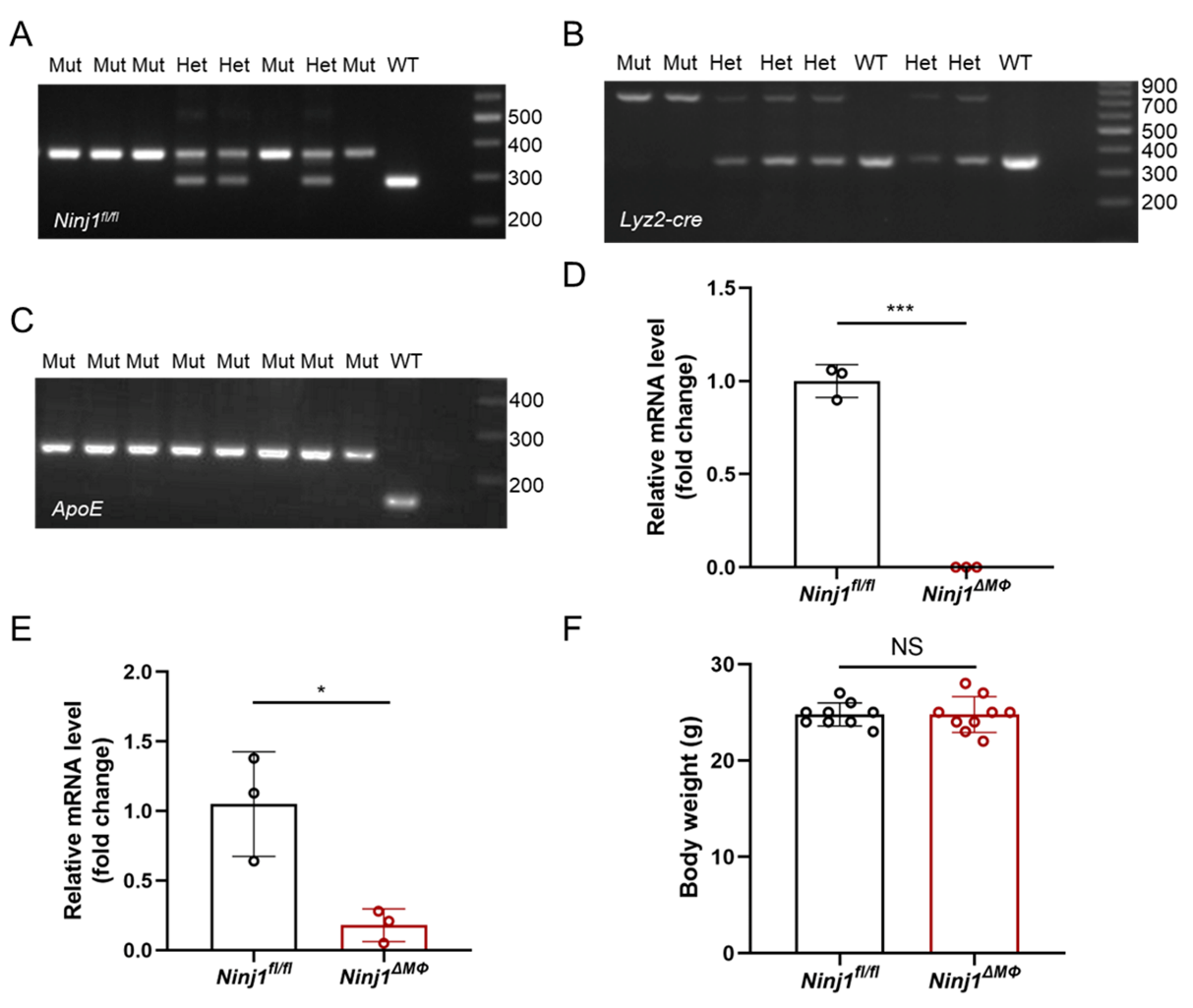


**Figure S2. Genotype identification of transgenic mice. (A)** Genotype identification using murine tail DNA by polymerase chain reaction (PCR) and agarose-gel electrophoresis. PCR screening of *Ninj1* showed a band of 357bp for the floxed allele and 267bp for the wild-type allele. **(B)** Genotype identification using murine tail DNA by PCR and agarose-gel electrophoresis. PCR screening of *Lyz2-cre* showed a band of 750bp for the mutant allele and 350bp for the wild-type allele. **(C)** Genotype identification using murine tail DNA by PCR and agarose-gel electrophoresis. PCR screening of *ApoE* showed a band of 254bp for the mutant allele and 155bp for the wild-type allele. **(D)** Quantification of *Ninj1* mRNA expression level by quantitative PCR (qPCR) in murine bone marrow-derived macrophages (BMDMs). Data were analyzed by the Student’s *t*-test; n = 3. ****P* < 0.001. **(E)** Quantification of *Ninj1* mRNA expression level by qPCR in murine spleen tissues. Data were analyzed by the Student’s *t*-test; n = 3. **P* < 0.05. **(F)** Body weight of 12-week-old male *Ninj1^fl/fl^* and *Ninj1^ΔMΦ^* mice. Data were analyzed by the Student’s *t*-test; n = 10. NS indicates *P* > 0.05.

**
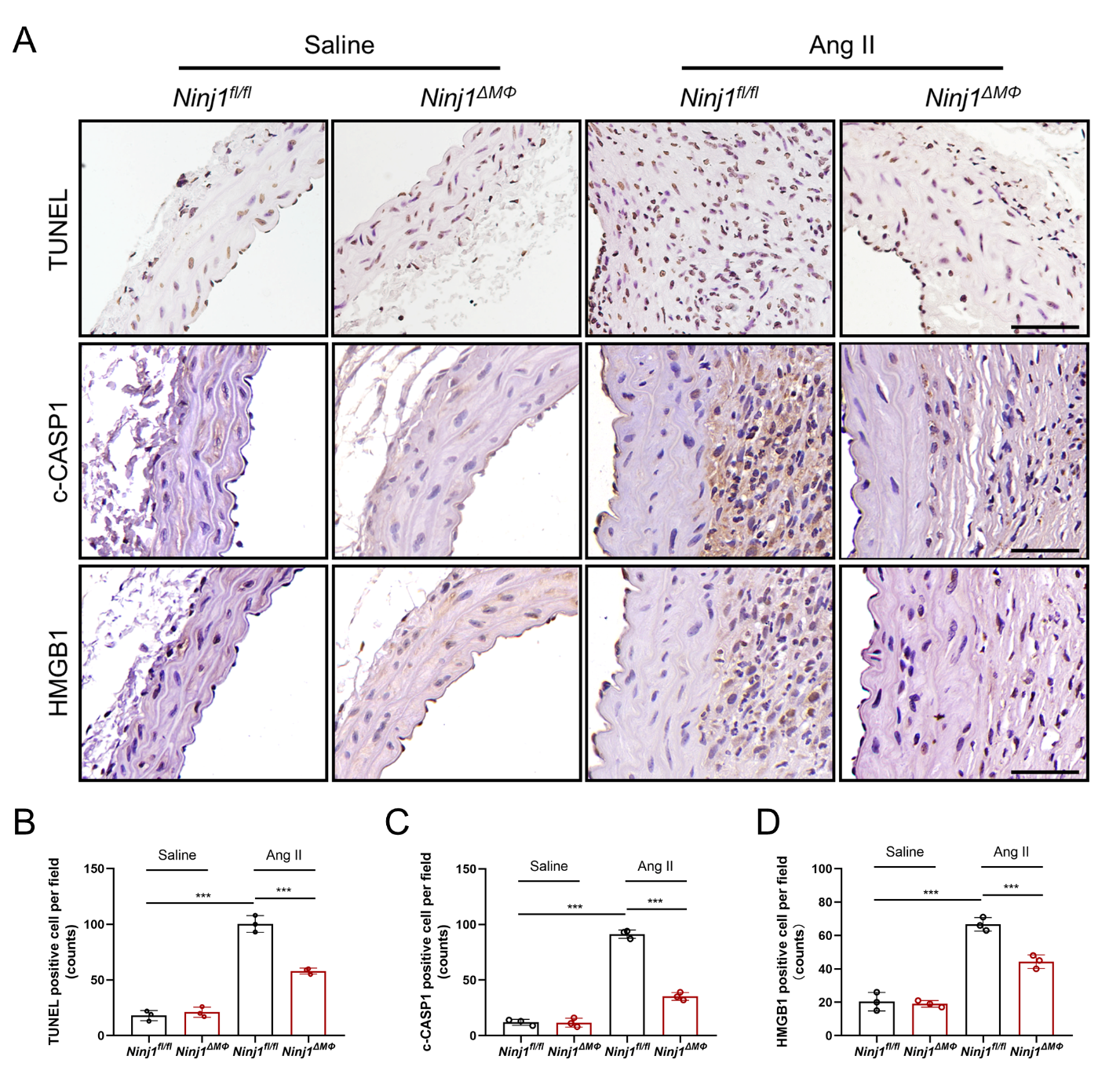
**

**Figure S3. Macrophage NINJ1 deficiency suppresses cell apoptosis, pyroptosis, and necrosis in abdominal aortic tissues.** **(A)** Representative images of TdT-mediated dUTP nick-end labeling (TUNEL), cleaved caspase-1 (c-CASP1), and high mobility group box 1 (HMGB1) staining through immunohistochemistry in murine abdominal aortic tissues from the indicated groups. Scale bar = 50 μm. **(B)** Quantification of the number of TUNEL-positive cells in panel A. Data were analyzed by two-way ANOVA followed by the Bonferroni post hoc test; n = 3. ****P* < 0.001. **(C)** Quantification of the number of c-CASP1-positive cells in panel A. Data were analyzed by two-way ANOVA followed by the Bonferroni post hoc test; n = 3. ****P* < 0.001. **(D)** Quantification of the number of HMGB1-positive cells in panel A. Data were analyzed by two-way ANOVA followed by the Bonferroni post hoc test; n = 3. ****P* < 0.001.


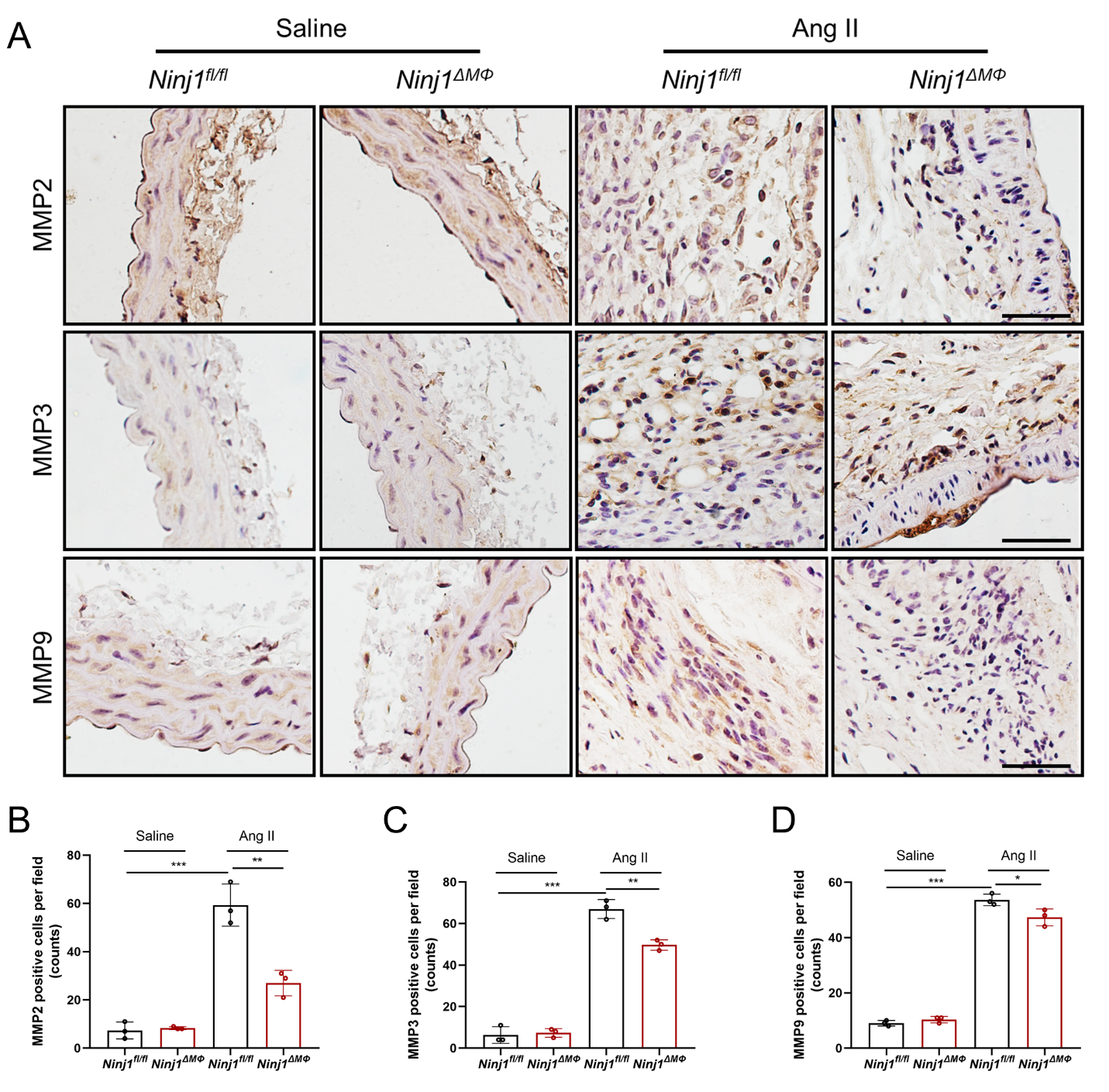


**Figure S4. Macrophage NINJ1 deficiency suppresses the expression levels of matrix metalloproteinases (MMPs) in abdominal aortic tissues. (A)** The representative images of staining of MMP2, MMP3, and MMP9 by immunohistochemistry in murine abdominal aortic tissues from the indicated groups. Scale bar = 50 μm. **(B)** Quantification of the number of MMP2-positive cells in the indicated groups. Data were analyzed by two-way ANOVA followed by the Bonferroni post hoc test; n = 3. ***P* < 0.01; ****P* < 0.001. **(C)** Quantification of the numbers of MMP3-positive cells in the indicated groups. Data were analyzed by two-way ANOVA followed by the Bonferroni post hoc test; n = 3. ***P* < 0.01; ****P* < 0.001. **(D)** Quantification of the numbers of MMP9-positive cells in the indicated groups. Data were analyzed by two-way ANOVA followed by the Bonferroni post hoc test; n = 3. **P* < 0.05; ****P* < 0.001.


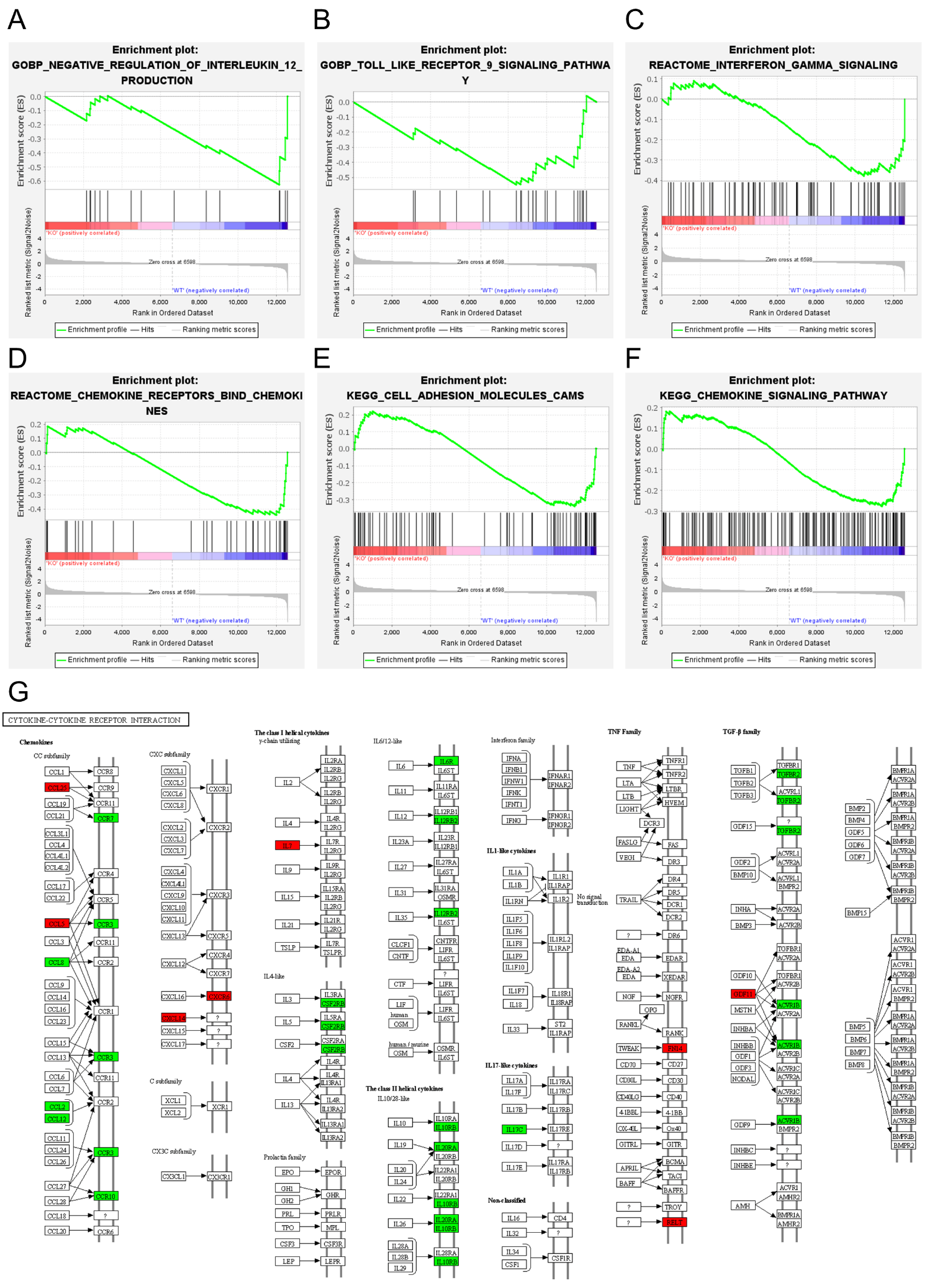


**Figure S5. NINJ1 perturbs inflammation, cell adhesion, and chemokine pathways in macrophages.** **(A)** The pathway associated with the negative regulation of interleukin-12 production (GO: 0045083) exhibited a significant downregulation in bone marrow-derived macrophages (BMDMs) obtained from *Ninj1^ΔMΦ^* mice. **(B)** The Toll-like receptor 9 signaling pathway (GO: 0034162) is significantly downregulated in BMDMs from *Ninj1^ΔMΦ^* mice. **(C)** The interferon-gamma signaling pathway (Reactome: R-HSA-877300) was significantly downregulated in BMDMs from *Ninj1^ΔMΦ^* mice. **(D)** The chemokine receptors bind chemokine pathway (Reactome: R-HSA-380108) was significantly downregulated in BMDMs from *Ninj1^ΔMΦ^* mice. **(E)** The pathway of cell adhesion molecules (KEGG: mmu04514) was significantly downregulated in BMDMs from *Ninj1^ΔMΦ^* mice. **(F)** The chemokine signaling pathway (KEGG: mmu04062) was significantly downregulated in BMDMs from *Ninj1^ΔMΦ^* mice. **(G)** The intact KEGG pathway map of cytokine-cytokine receptor interaction.


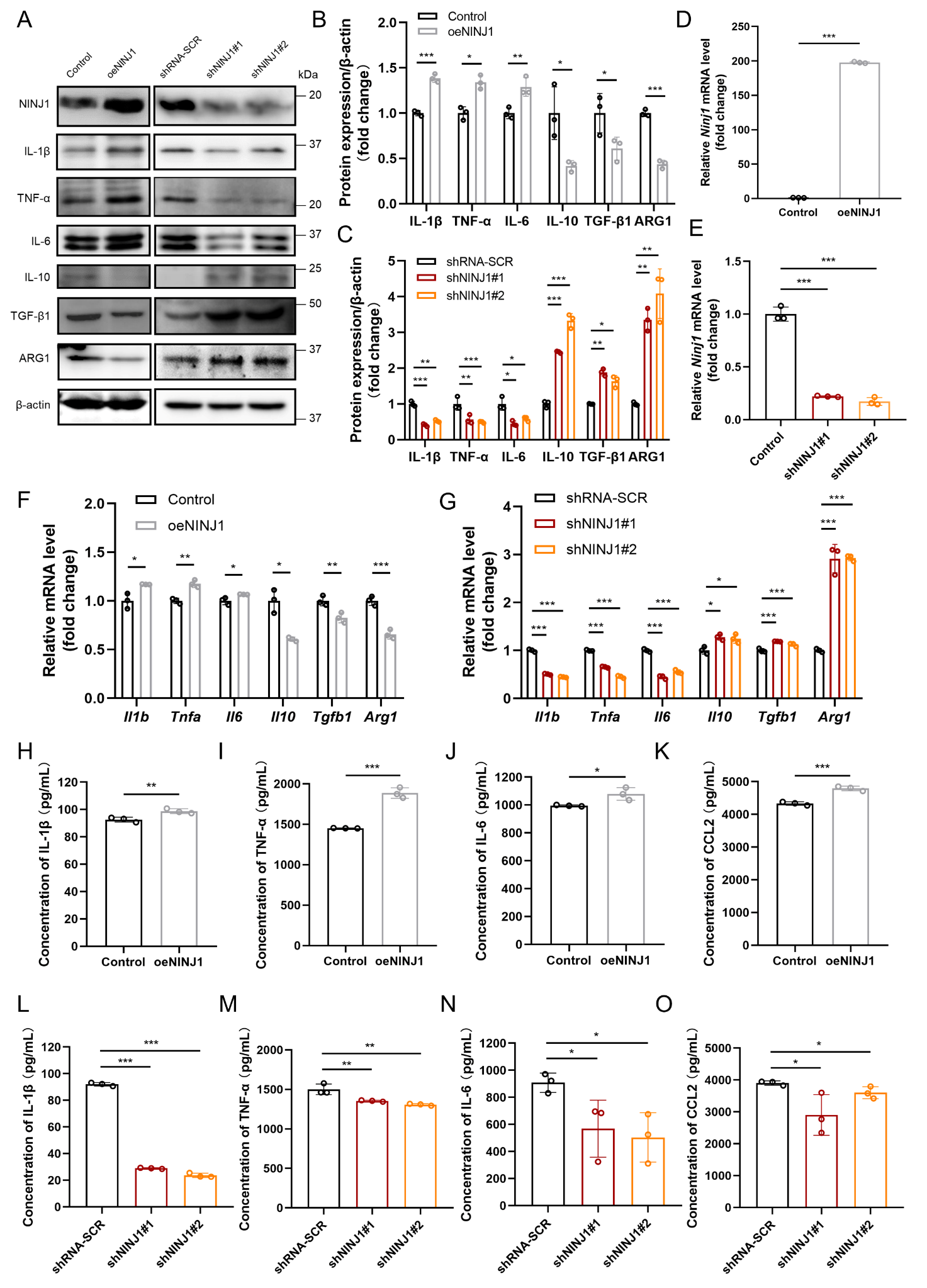


**Figure S6. NINJ1 upregulates the pro-inflammatory phenotype of macrophages.** **(A)** The levels of NINJ1, IL-1β, TNF-α, IL-6, IL-10, TGF-β1, and ARG1 proteins were measured by Western blotting in NINJ1 overexpression or NINJ1 knockdown Raw264.7 cells. **(B)** and **(C)** Quantification of NINJ1, IL-1β, TNF-α, IL-6, IL-10, TGF-β1, and ARG1 protein expression levels in panel A. The level of β-actin protein was used for normalization. Data were analyzed by the Student’s *t*-test; n = 3. **P* < 0.05; ***P* < 0.01; ****P* < 0.001. **(D)** and **(E)** Quantification of *Ninj1* mRNA expression level by quantitative polymerase chain reaction (qPCR) in NINJ1 overexpression or NINJ1 knockdown Raw264.7 cells. Data were analyzed by the Student’s *t*-test; n = 3. ****P* < 0.001. **(F)** and **(G)** Quantification of *Il1b, Tnfa, Il6,* *Il10, Tgfb1,* and *Arg1* mRNA expression levels by qPCR in the indicated groups. Data were analyzed by the Student’s *t*-test; n = 3. **P* < 0.05; ***P* < 0.01; ****P* < 0.001. **(H)** IL-1β, **(I)** TNF-α, **(J)** IL-6, and **(K)** CCL2 protein concentrations were measured by ELISA in cell supernatant from NINJ1 overexpression Raw264.7 cells. Data were analyzed by the Student’s *t*-test; n = 3. **P* < 0.05; ***P* < 0.01; ****P* < 0.001. **(L)** IL-1β, **(M)** TNF-α, **(N)** IL-6, and **(O)** CCL2 protein concentrations in cell supernatant from NINJ1 knockdown Raw264.7 cells measured by ELISA. Data were analyzed by the Student’s *t*-test; n = 3. **P* < 0.05; ***P* < 0.01; ****P* < 0.001.


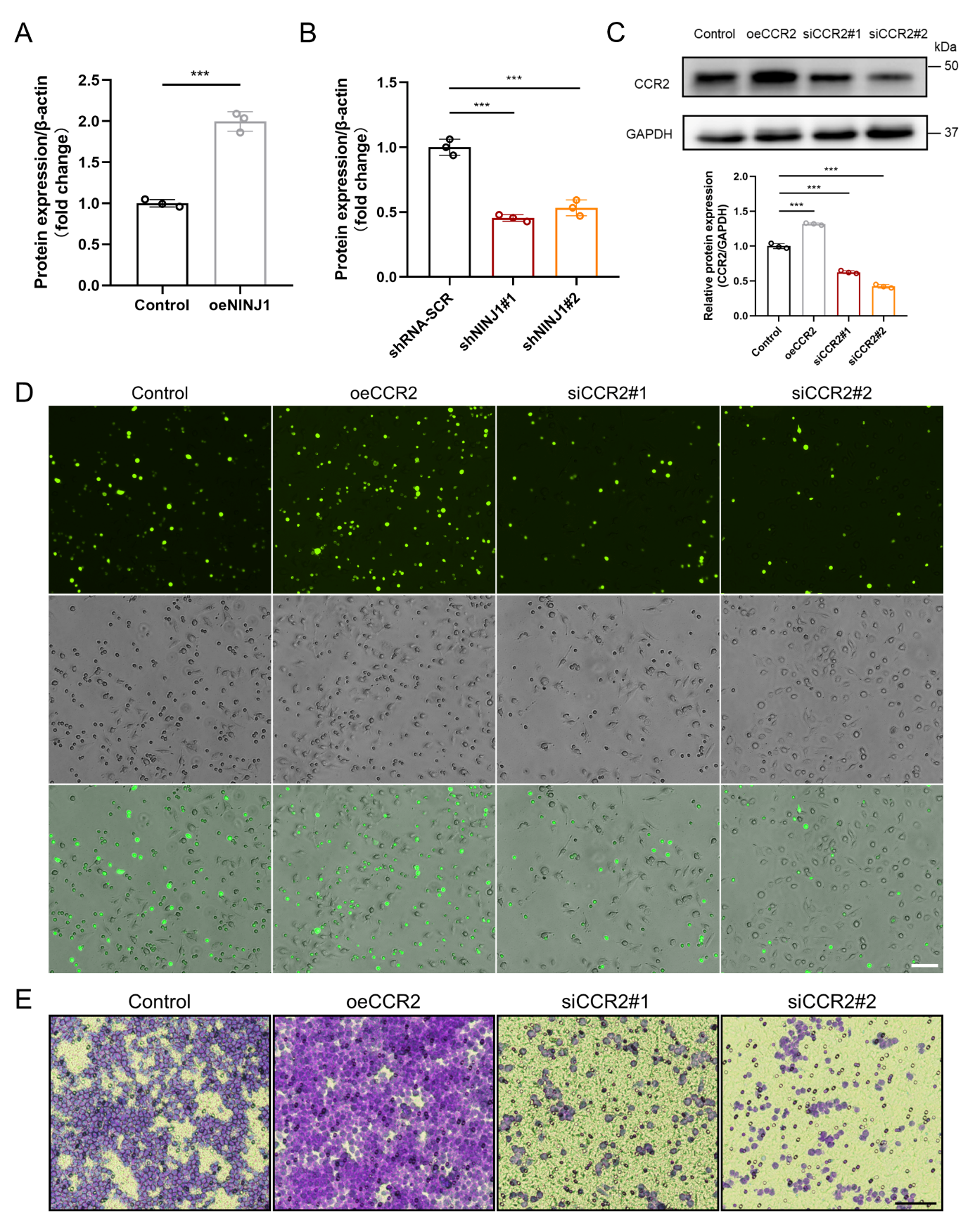


**Figure S7. CCR2 positively regulates adhesion and trans-endothelial migration of macrophages.** **(A)** and **(B)** Quantification of CCR2 protein expression level by Western blotting in NINJ1 overexpression or NINJ1 knockdown Raw264.7 cells. The level of β-actin protein was used for normalization. Data were analyzed by the Student’s *t*-test; n = 3. ****P* < 0.001. **(C)** The CCR2 protein level was measured by Western blotting in CCR2 knockdown and CCR2 overexpression Raw264.7 cells. The GAPDH protein level was used for normalization. Data were analyzed by the Student’s *t*-test; n = 3. ****P* < 0.001. **(D)** Representative images of cell adhesion assay in the indicated groups. Raw 264.7 cells were pre-stained with fluorescent probe BCECF-AM (green). Scale bar = 10 μm. **(E)** Representative images of transwell migration assay in the indicated groups. Scale bar = 100 μm. Raw 264.7 cells were stained with crystal violet.


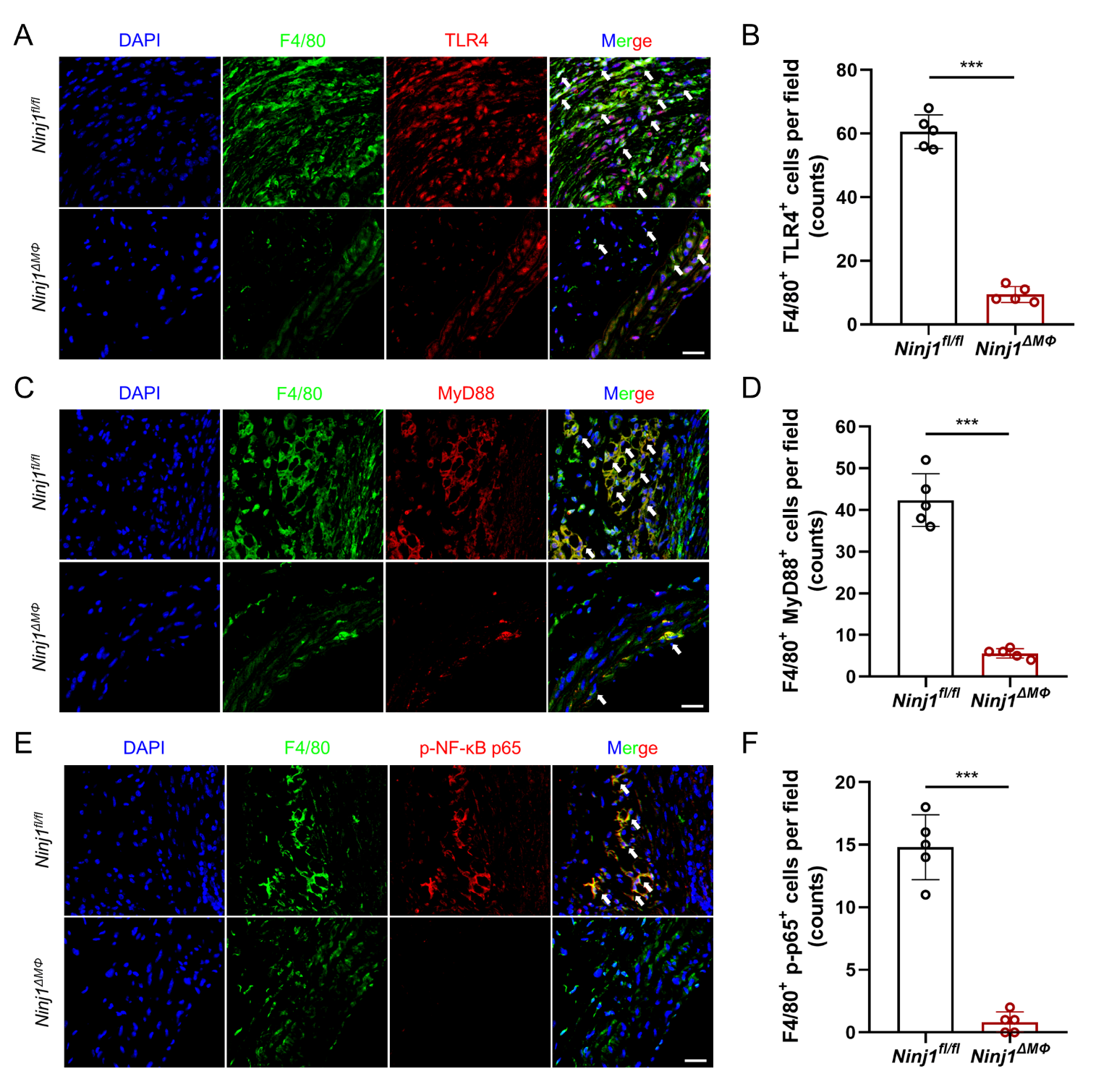


**Figure S8. Macrophage NINJ1 deficiency downregulates the TLR4/MyD88/NF-κB pathway in vivo. (A)** and **(B)** Immunofluorescence staining for F4/80 (green) and TLR4 (red) in murine AAA tissues. Nuclei were stained by DAPI (blue). White arrows indicate F4/80^+^/ TLR4^+^ cells. Scale bar = 25 μm. Data were analyzed by the Student’s *t*-test; n = 5. ****P* < 0.001. **(C)** and **(D)** Immunofluorescence staining for F4/80 (green) and MyD88 (red) in murine AAA tissues. Nuclei were stained by DAPI (blue). White arrows indicate F4/80^+^/ MyD88^+^ cells. Scale bar = 25 μm. Data were analyzed by the Student’s *t*-test; n = 5. ****P* < 0.001. **(E)** and **(F)** Immunofluorescence staining for F4/80 (green) and phospho-NF-κB p65 (red) in murine AAA tissues. Nuclei were stained by DAPI (blue). White arrows indicate F4/80^+^/ p-p65^+^ cells. Scale bar = 25 μm. Data were analyzed by the Student’s *t*-test; n = 5. ****P* < 0.001.


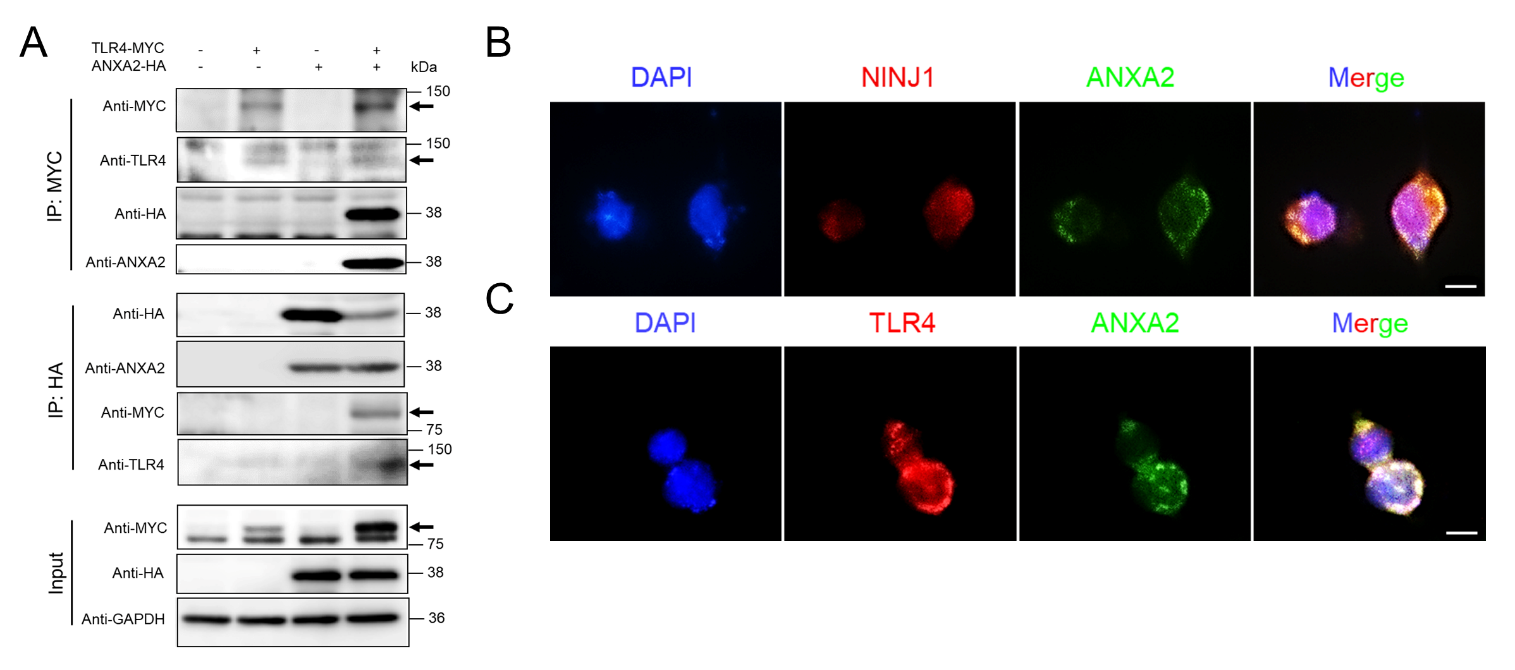


**Figure S9. NINJ1 and TLR4 are co-localized with ANXA2 in the cell membrane. (A)** Verification of the interaction between TLR4 and ANXA2. HEK293T cells were transfected with MYC-tagged TLR4 and HA-tagged ANXA2 plasmids. TLR4 protein was immunoprecipitated by anti-MYC antibody combined with protein A/G agarose resin, and ANXA2 protein was immunoprecipitated by anti-HA antibody combined with protein A/G agarose resin. The indicated protein expression levels in the precipitation were detected by Western blotting. The arrows indicate target bands. **(B)** Representative images of co-immunofluorescence staining of AXNA2 (green) and NINJ1 (red) in HEK293T cells. HEK293T cells were transfected with Flag-tagged NINJ1 and HA-tagged ANXA2 plasmids for 24 h. The nuclei were stained with DAPI (blue). Scale bar = 1 μm. **(C)** Representative images of co-immunofluorescence staining of AXNA2 (green) and TLR4 (red) in HEK293T cells. HEK293T cells were transfected with MYC-tagged TLR4 and HA-tagged ANXA2 plasmids for 24 h. The nuclei were stained with DAPI (blue). Scale bar = 1 μm.

**Supplementary Tables**

**Table S1. Characteristics of participants enrolled in the ELISA study.**

| **Variable** | **Control (n = 10)** | **AAA (n = 14)** | ***P*-value** |
| --- | --- | --- | --- |
| Male sex | 7 (70.0) | 12 (85.7) | 0.615 |
| Age | 65.50 ± 12.75 | 73.00 ± 8.95 | 0.130 |
| Body mass index | 24.46 ± 3.39 | 25.59 ± 3.26 | 0.422 |
| Smoker | 1 (10.0) | 3 (21.4) | 0.615 |
| Hypertension | 6 (60.0) | 11 (78.6) | 0.393 |
| Diabetes mellitus | 1 (10.0) | 5 (35.7) | 0.341 |
| Peripheral arterial disease | 2 (20.0) | 8 (57.1) | 0.104 |
| COPD | 0 (0) | 0 (0) | 1.000 |
| Ischemic heart disease | 0 (0) | 4 (28.6) | 0.114 |
| Stroke | 1 (10.0) | 5 (35.7) | 0.341 |
| Chronic kidney disease | 0 (0) | 4 (28.6) | 0.114 |
| SBP (mmHg) | 143.20 ± 22.33 | 138.64 ± 14.52 | 0.563 |
| DBP (mmHg) | 83.00 ± 11.63 | 79.07 ± 9.39 | 0.383 |
| Heart rate (bpm) | 82.70 ± 9.65 | 78.93 ± 7.88 | 0.317 |
| Total cholesterol (mmol L^-1^) | 4.65 ± 1.28 | 4.96 ± 1.27 | 0.622 |
| Triglycerides (mmol L^-1^) | 1.67 ± 0.81 | 1.46 ± 0.52 | 0.523 |
| HDL (mmol L^-1^) | 1.22 ± 0.40 | 1.00 ± 0.16 | 0.135 |
| LDL (mmol L^-1^) | 2.88 ± 0.92 | 3.57 ± 1.15 | 0.185 |
| Fasting glucose (mmol L^-1^) | 5.43 ± 1.95 | 6.55 ± 3.71 | 0.436 |
| eGFR (mL min^-1^ 1.73m^-2^) | 97.60 ± 9.57 | 77.71 ± 24.83 | 0.026 |
| D-dimer (mg L^-1^) | 4.73 ± 7.24 | 3.78 ± 5.37 | 0.749 |
| Antihypertensive drugs | 6 (60.0) | 10 (71.4) | 0.673 |
| Anticoagulant drugs | 10 (100.0) | 14 (100.0) | 1.000 |
| Lipid-lowering drugs | 0 (0) | 6 (42.9) | 0.024 |

Data were expressed as Mean ± SD or n (%). AAA, abdominal aortic aneurysm; COPD, chronic obstructive pulmonary disease; SBP, systolic blood pressure; DBP, diastolic blood pressure; HDL, high-density lipoprotein; LDL, low-density lipoprotein; eGFR, estimated glomerular filtration rate.

**Table S2. Primary antibodies used in Western blot assay.**

| **Target Antigen** | **Vendor or Source** | **Catalog #** | **Working Concentration** | **Persistent ID / ULR** |
| --- | --- | --- | --- | --- |
| NINJ1 | BD | 610776 | 0.25 μg mL^-1^ | https://www.bdbiosciences.com/zh-cn/products/reagents/microscopy-imaging-reagents/immunofluorescence-reagents/purified-mouse-anti-ninjurin.610776 |
| NINJ1 | Invitrogen | PA5-95755 | 0.44 μg mL^-1^ | https://www.thermofisher.cn/cn/zh/antibody/product/Ninjurin-1-Antibody-Polyclonal/PA5-95755 |
| F4/80 | Abcam | ab6640 | 10 μg mL^-1^ | https://www.abcam.cn/products/primary-antibodies/f480-antibody-cia3-1-macrophage-marker-ab6640 |
| CD31 | Abcam | ab28364 | 0.26 μg mL^-1^ | https://www.abcam.cn/products/primary-antibodies/cd31-antibody-ab28364 |
| α-SMA | Abcam | ab5694 | 1 μg mL^-1^ | https://www.abcam.cn/products/primary-antibodies/alpha-smooth-muscle-actin-antibody-ab5694 |
| β-actin | Proteintech | 66009-1-Ig | 0.05 μg mL^-1^ | https://www.ptgcn.com/products/Pan-Actin-Antibody-66009-1-Ig |
| INOS | CST | 13120S | 0.1 μg mL^-1^ | https://www.cellsignal.cn/products/primary-antibodies/inos-d6b6s-rabbit-mab/13120?site-search-type=Products&N=4294956287&Ntt=13120s&fromPage=plp&_requestid=884129 |
| ARG1 | Novus Biologicals | NBP1-32731 | 1 μg mL^-1^ | https://www.novusbio.com/products/arginase-1-arg1-liver-arginase-antibody_nbp1-32731 |
| CD68 | Proteintech | 28058-1-AP | 0.4 μg mL^-1^ | https://www.ptgcn.com/products/Cd68-Antibody-28058-1-AP |
| CD206 | Abcam | ab64693 | 1 μg mL^-1^ | https://www.abcam.cn/products/primary-antibodies/mannose-receptor-antibody-ab64693 |
| MMP2 | Abcam | ab92536 | 7.4 μg mL^-1^ | https://www.abcam.cn/products/primary-antibodies/mmp2-antibody-epr1184-ab92536 |
| MMP3 | Abcam | ab52915 | 1.54 μg mL^-1^ | https://www.abcam.cn/products/primary-antibodies/mmp3-antibody-ep1186y-ab52915 |
| MMP9 | Abcam | ab283575 | 12.5 μg mL^-1^ | https://www.abcam.cn/products/primary-antibodies/mmp9-antibody-rm1020-ab283575 |
| c-CASP1 | Invitrogen | PA5-38099 | 10 μg mL^-1^ | https://www.thermofisher.cn/cn/zh/antibody/product/Caspase-1-Cleaved-Asp210-Antibody-Polyclonal/PA5-38099 |
| HMGB1 | Invitrogen | MA5-31967 | 0.2 μg mL^-1^ | https://www.thermofisher.cn/cn/zh/antibody/product/HMGB1-Antibody-clone-SA39-03-Recombinant-Monoclonal/MA5-31967 |
| IL-1β | Proteintech | 26048-1-AP | 0.85 μg mL^-1^ | https://www.ptgcn.com/products/Il1b-Antibody-26048-1-AP |
| IL-6 | SAB | 41739 | 1 μg mL^-1^ | https://www.sabbiotech.cn/g-15422-IL-6-Polyclonal-Antibody-41739 |
| TNF-α | Proteintech | 17590-1-AP | 0.6 μg mL^-1^ | https://www.ptgcn.com/products/TNFA-Antibody-17590-1-AP |
| TGF-β1 | Proteintech | 21898-1-AP | 0.6 μg mL^-1^ | https://www.ptgcn.com/products/TGF-beta-1-Antibody-21898-1-AP |
| IL-10 | Proteintech | 60269-1-Ig | 0.28 μg mL^-1^ | https://www.ptgcn.com/products/IL10-Antibody-60269-1-Ig |
| ANXA2 | SAB | 40592 | 1 μg mL^-1^ | https://www.sabbiotech.cn/g-14275-Annexin-II-Polyclonal-Antibody-40592 |
| TLR4 | Proteintech | 66350-1-AP | 1.7 μg mL^-1^ | https://www.ptgcn.com/products/TLR4-Antibody-66350-1-Ig |
| MyD88 | CST | 4283S | 0.1 μg mL^-1^ | https://www.cellsignal.cn/products/primary-antibodies/myd88-d80f5-rabbit-mab/4283?site-search-type=Products&N=4294956287&Ntt=4283&fromPage=plp&_requestid=884449 |
| Phospho-NF-κB p65 | CST | 3033S | 0.1 μg mL^-1^ | https://www.cellsignal.cn/products/primary-antibodies/phospho-nf-kb-p65-ser536-93h1-rabbit-mab/3033?site-search-type=Products&N=4294956287&Ntt=3033s&fromPage=plp&_requestid=884583 |
| NF-κB p65 | CST | 8242S | 0.1 μg mL^-1^ | https://www.cellsignal.cn/products/primary-antibodies/nf-kb-p65-d14e12-xp-rabbit-mab/8242?site-search-type=Products&N=4294956287&Ntt=8242s&fromPage=plp&_requestid=884755 |
| HA | Proteintech | 66006-2-Ig | 0.12 μg mL^-1^ | https://www.ptgcn.com/products/HA-Tag-Antibody-66006-2-Ig |
| MYC | Proteintech | 60003-2-Ig | 0.5 μg mL^-1^ | https://www.ptgcn.com/products/MYC-Antibody-60003-2-Ig |
| Flag | Proteintech | 20543-1-AP | 0.03 μg mL^-1^ | https://www.ptgcn.com/products/Flag-Tag-Antibody-20543-1-AP |
| CCR2 | Proteintech | 16153-1-AP | 0.6 μg mL^-1^ | https://www.ptgcn.com/products/CCR2a-specific-Antibody-16153-1-AP |
| GAPDH | Proteintech | 60004-1-Ig | 0.02 μg mL^-1^ | https://www.ptgcn.com/products/GAPDH-Antibody-60004-1-Ig |

**Table S3: Oligonucleotide sequences used in the study.**

| **Primer** | **Sequence** |
| --- | --- |
| shNINJ1#1 | GCCCATCAATGTAAACCATTACTTCCTGTCAGATAATGGTTTACATTGATGGGC |
| shNINJ1#2 | GCAATGATTTCGCCTTCTTCGCTTCCTGTCAGACGAAGAAGGCGAAATCATTGC |
| siCCR2#1 | UGCUAAACGUCUCUGCAAA |
| siCCR2#2 | UUUGCAGAGACGUUUAGCA |

**Table S4: Primer sequences used in RT-qPCR assay.**

| **Gene** | **Primer Sequence (5'-3')** |
| --- | --- |
| *Ninj1* | Forward: CCATTATGCCAACAAGAAGAGCG |
|  | Reverse: TGAAGATGTTGACCACGACGAT |
| *Il1b* | Forward: TGCCACCTTTTGACAGTGATG |
|  | Reverse: CATCTCGGAGCCTGTAGTGC |
| *Tnfa* | Forward: TGGAACTGGCAGAAGAGGCAC |
|  | Reverse: AGGGTCTGGGCCATAGAACTGA |
| *Ccl2* | Forward: GCTCAAGAGAGAGGTCTGTG |
|  | Reverse: AGATTTACGGGTCAACTTCA |
| *Mmp2* | Forward: CCCCGGTTTCCCTAAGCT |
|  | Reverse: CACGCTCTTGAGACTTTGGTTC |
| *Mmp3* | Forward: CTCCACAGACTTGTCCCGTTT |
|  | Reverse: AGTCCTGAGAGATTTGCGCC |
| *Mmp9* | Forward: AAAGACGACATAGACGGCATCC |
|  | Reverse: TCAGAAGAGCCCGCAGTAGG |
| *Cd163* | Forward: TCTGCTGTCACTAACGCTCCT |
|  | Reverse: CAAACCACGGACACTTCATTCA |
| *Cd206* | Forward: GCTGGCGAGCATCAAGAGTA |
|  | Reverse: AGGAAACGGGAGAACCATCAC |
| *Il6* | Forward: TTCTTGGGACTGATGCTGGTG |
|  | Reverse: CACAACTCTTTTCTCATTTCCACGA |
| *Il10* | Forward: TTACCTGGTAGAAGTGATGCCC |
|  | Reverse: GACACCTTGGTCTTGGAGCTTA |
| *Tgfb1* | Forward: AACAATTCCTGGCGTTACCTT |
|  | Reverse: TCGAAAGCCCTGTATTCCGTCT |
| *Arg1* | Forward: CATATCTGCCAAAGACATCGTG |
|  | Reverse: GACATCAAAGCTCAGGTGAATC |
| *Ccr2* | Forward: AGCCTTGTCATAAAACCAGTGTG |
|  | Reverse: GGTAATGTGAGCAGGAAGAGCA |
| *Gapdh* | Forward: CCTCGTCCCGTAGACAAAATG |
|  | Reverse: TGAGGTCAATGAAGGGGTCGT |

**Table S5. NINJ1-interacted proteins identified by immunoprecipitation-mass spectrometry.**

| **Gene Name** | **Uniprot Entry** | **Protein Name** |
| --- | --- | --- |
| SPRR2D | P22532 | Small proline-rich protein 2D |
| CEBPZOS | A8MTT3 | Protein CEBPZOS |
| MRPS18B | Q9Y676 | 28S ribosomal protein S18b, mitochondrial |
| TMEM184B | Q9Y519 | Transmembrane protein 184B |
| GIGYF2 | Q6Y7W6 | GRB10-interacting GYF protein 2 |
| ARPC5 | O15511 | Actin-related protein 2/3 complex subunit 5 |
| DDX10 | Q13206 | Probable ATP-dependent RNA helicase DDX10 |
| ST3GAL1 | Q11201 | CMP-N-acetylneuraminate-beta-galactosamide-alpha-2,3-sialyltransferase 1 |
| USO1 | O60763 | General vesicular transport factor p115 |
| PRAF2 | O60831 | PRA1 family protein 2 |
| TRMU | O75648 | Mitochondrial tRNA-specific 2-thiouridylase 1 |
| SPATA5 | Q8NB90 | Ribosome biogenesis protein SPATA5 |
| VDAC3 | Q9Y277 | Voltage-dependent anion-selective channel protein 3 |
| TRIP6 | Q15654 | Thyroid receptor-interacting protein 6 |
| ECT2 | Q9H8V3 | Protein ECT2 |
| DNAH17 | Q9UFH2 | Dynein axonemal heavy chain 17 |
| EDC3 | Q96F86 | Enhancer of mRNA-decapping protein 3 |
| NUMA1 | Q14980 | Nuclear mitotic apparatus protein 1 |
| ELP3 | Q9H9T3 | Elongator complex protein 3 |
| GART | P22102 | Trifunctional purine biosynthetic protein adenosine-3 |
| RSL1D1 | O76021 | Ribosomal L1 domain-containing protein 1 |
| PDCD4 | Q53EL6 | Programmed cell death protein 4 |
| HSPA14 | Q0VDF9 | Heat shock 70 kDa protein 14 |
| PRMT3 | O60678 | Protein arginine N-methyltransferase 3 |
| FSTL1 | Q12841 | Follistatin-related protein 1 |
| SEC63 | Q9UGP8 | Translocation protein SEC63 homolog |
| ACAA2 | P42765 | 3-ketoacyl-CoA thiolase, mitochondrial |
| SF1 | Q15637 | Splicing factor 1 |
| NDUFB10 | O96000 | NADH dehydrogenase [ubiquinone] 1 beta subcomplex subunit 10 |
| BLMH | Q13867 | Bleomycin hydrolase |
| FHL1 | Q13642 | Four and a half LIM domains protein 1 |
| NOX3 | Q9HBY0 | NADPH oxidase 3 |
| LGALS1 | P09382 | Galectin-1 |
| MRPS23 | Q9Y3D9 | 28S ribosomal protein S23, mitochondrial |
| NSUN6 | Q8TEA1 | tRNA (cytosine(72)-C(5))-methyltransferase NSUN6 |
| GEMIN5 | Q8TEQ6 | Gem-associated protein 5 |
| MRPL11 | Q9Y3B7 | 39S ribosomal protein L11, mitochondrial |
| HTT | P42858 | Huntingtin |
| DNAH8 | Q96JB1 | Dynein axonemal heavy chain 8 |
| NDUFA4 | O00483 | Cytochrome c oxidase subunit NDUFA4 |
| MKRN2 | Q9H000 | E3 ubiquitin-protein ligase makorin-2 |
| AKR1B1 | P15121 | Aldo-keto reductase family 1 member B1 |
| MAP7D1 | Q3KQU3 | MAP7 domain-containing protein 1 |
| RNGTT | O60942 | mRNA-capping enzyme |
| GNG5 | P63218 | Guanine nucleotide-binding protein G(I)/G(S)/G(O) subunit gamma-5 |
| NUCB2 | P80303 | Nucleobindin-2 |
| CSNK2A1 | P68400 | Casein kinase II subunit alpha |
| NOTCH2 | Q04721 | Neurogenic locus notch homolog protein 2 |
| ABCB10 | Q9NRK6 | ATP-binding cassette sub-family B member 10, mitochondrial |
| NOD1 | Q9Y239 | Nucleotide-binding oligomerization domain-containing protein 1 |
| BMS1 | Q14692 | Ribosome biogenesis protein BMS1 homolog |
| CAND1 | Q86VP6 | Cullin-associated NEDD8-dissociated protein 1 |
| MYH14 | Q7Z406 | Myosin-14 |
| CMSS1 | Q9BQ75 | Protein CMSS1 |
| SACS | Q9NZJ4 | Sacsin |
| LRRC47 | Q8N1G4 | Leucine-rich repeat-containing protein 47 |
| PCID2 | Q5JVF3 | PCI domain-containing protein 2 |
| MBNL1 | Q9NR56 | Muscleblind-like protein 1 |
| CKMT1B | P12532 | Creatine kinase U-type, mitochondrial |
| NIPSNAP1 | Q9BPW8 | Protein NipSnap homolog 1 |
| MTCH2 | Q9Y6C9 | Mitochondrial carrier homolog 2 |
| KIF2C | Q99661 | Kinesin-like protein KIF2C |
| VN1R5 | Q7Z5H4 | Vomeronasal type-1 receptor 5 |
| FMR1 | Q06787 | Fragile X messenger ribonucleoprotein 1 |
| SRPK2 | P78362 | SRSF protein kinase 2 |
| VDAC1 | P21796 | Voltage-dependent anion-selective channel protein 1 |
| ZFR | Q96KR1 | Zinc finger RNA-binding protein |
| NOC2L | Q9Y3T9 | Nucleolar complex protein 2 homolog |
| UQCRC2 | P22695 | Cytochrome b-c1 complex subunit 2, mitochondrial |
| LZTS2 | Q9BRK4 | Leucine zipper putative tumor suppressor 2 |
| NMD3 | Q96D46 | 60S ribosomal export protein NMD3 |
| NDUFS3 | O75489 | NADH dehydrogenase [ubiquinone] iron-sulfur protein 3, mitochondrial |
| RBM15 | Q96T37 | RNA-binding protein 15 |
| PARP1 | P09874 | Poly [ADP-ribose] polymerase 1 |
| FN1 | P02751 | Fibronectin |
| VDAC2 | P45880 | Voltage-dependent anion-selective channel protein 2 |
| ANXA2 | P07355 | Annexin A2 |
| PRDX6 | P30041 | Peroxiredoxin-6 |
| CPNE3 | O75131 | Copine-3 |
| SNRPD1 | P62314 | Small nuclear ribonucleoprotein Sm D1 |
| ACP1 | P24666 | Low molecular weight phosphotyrosine protein phosphatase |
| STON2 | Q8WXE9 | Stonin-2 |
| DHX36 | Q9H2U1 | ATP-dependent DNA/RNA helicase DHX36 |
| RPS5 | P46782 | 40S ribosomal protein S5 |
| XPO5 | Q9HAV4 | Exportin-5 |
| MCM5 | P33992 | DNA replication licensing factor MCM5 |
| MCM7 | P33993 | DNA replication licensing factor MCM7 |
| SNX27 | Q96L92 | Sorting nexin-27 |
| RPL36A | P83881 | 60S ribosomal protein L36a |
| DAD1 | P61803 | Dolichyl-diphosphooligosaccharide--protein glycosyltransferase subunit DAD1 |
| RTN3 | O95197 | Reticulon-3 |
| DHX29 | Q7Z478 | ATP-dependent RNA helicase DHX29 |
| SMARCA5 | O60264 | SWI/SNF-related matrix-associated actin-dependent regulator of chromatin subfamily A member 5 |
| SPTBN2 | O15020 | Spectrin beta chain, non-erythrocytic 2 |
| ILF3 | Q12906 | Interleukin enhancer-binding factor 3 |
| MRPS12 | O15235 | 28S ribosomal protein S12, mitochondrial |
| MRI1 | Q9BV20 | Methylthioribose-1-phosphate isomerase |
| RRP1B | Q14684 | Ribosomal RNA processing protein 1 homolog B |
| PRKAG1 | P54619 | 5'-AMP-activated protein kinase subunit gamma-1 |
| HNRNPU | Q00839 | Heterogeneous nuclear ribonucleoprotein U |
| PLS3 | P13797 | Plastin-3 |
| KRT5 | P13647 | Keratin, type II cytoskeletal 5 |
| CDK4 | P11802 | Cyclin-dependent kinase 4 |
| C1orf35 | Q9BU76 | Multiple myeloma tumor-associated protein 2 |
| HSD17B12 | Q53GQ0 | Very-long-chain 3-oxoacyl-CoA reductase |
| GBAS | O75323 | Protein NipSnap homolog 2 |
| NARS | O43776 | Asparaginyl-tRNA synthetase |
| FAM208A | Q9UK61 | Protein TASOR |
| PHB | P35232 | Prohibitin 1 |
| KIAA0391 | O15091 | Mitochondrial ribonuclease P catalytic subunit |
| ERO1L | Q96HE7 | ERO1-like protein alpha |

**Table S6. TLR4 experimental interacted proteins screened from Integrated Interactions Database.**

| **Gene Name** | **Uniprot Entry** | **Protein Name** |
| --- | --- | --- |
| AHSG | P02765 | Alpha-2-HS-glycoprotein |
| ANPEP | P15144 | Aminopeptidase N |
| ANXA2 | P07355 | Annexin A2 |
| BCL10 | O95999 | B-cell lymphoma/leukemia 10 |
| BGN | P21810 | Biglycan |
| BIRC3 | Q13489 | Baculoviral IAP repeat-containing protein 3 |
| BTK | Q06187 | Tyrosine-protein kinase BTK |
| CAV1 | Q03135 | Caveolin-1 |
| CD14 | P08571 | Monocyte differentiation antigen CD14 |
| CD36 | P16671 | Platelet glycoprotein 4 |
| CIRBP | Q14011 | Cold-inducible RNA-binding protein |
| CNPY3 | Q9BT09 | Protein canopy homolog 3 |
| CNPY4 | Q8N129 | Protein canopy homolog 4 |
| DCN | P07585 | Decorin |
| DEFB4A | O15263 | Defensin beta 4A |
| DEFB4B | O15263 | Defensin beta 4B |
| EGFR | P00533 | Epidermal growth factor receptor |
| F2RL1 | P55085 | Proteinase-activated receptor 2 |
| HGS | O14964 | Hepatocyte growth factor-regulated tyrosine kinase substrate |
| HMGB1 | P09429 | High mobility group protein B1 |
| HSP90B1 | P14625 | Endoplasmin |
| HSPA14 | Q0VDF9 | Heat shock 70 kDa protein 14 |
| HSPA4 | P34932 | Heat shock 70 kDa protein 4 |
| HSPA8 | P11142 | Heat shock cognate 71 kDa protein |
| IKBKG | Q9Y6K9 | NF-kappa-B essential modulator |
| IL17RD | Q8NFM7 | Interleukin-17 receptor D |
| IRAK1 | P51617 | Interleukin-1 receptor-associated kinase 1 |
| IRAK2 | O43187 | Interleukin-1 receptor-associated kinase-like 2 |
| IRAK4 | Q9NWZ3 | Interleukin-1 receptor-associated kinase 4 |
| LY86 | O95711 | Lymphocyte antigen 86 |
| LY96 | Q9Y6Y9 | Lymphocyte antigen 96 |
| MAL | P21145 | Myelin and lymphocyte protein |
| MAPK8IP3 | Q9UPT6 | C-Jun-amino-terminal kinase-interacting protein 3 |
| MBL2 | P11226 | Mannose-binding protein C |
| MMP9 | P14780 | Matrix metalloproteinase-9 |
| MYD88 | Q99836 | Myeloid differentiation primary response protein MyD88 |
| MYH9 | P35579 | Myosin-9 |
| NEU1 | Q99519 | Sialidase-1 |
| NOX4 | Q9NPH5 | NADPH oxidase 4 |
| PIK3CD | O00329 | Phosphatidylinositol 4,5-bisphosphate 3-kinase catalytic subunit delta isoform |
| PPP4C | P60510 | Serine/threonine-protein phosphatase 4 catalytic subunit |
| PRKCZ | Q05513 | Protein kinase C zeta type |
| RIPK2 | O43353 | Receptor-interacting serine/threonine-protein kinase 2 |
| RNF216 | Q9NWF9 | E3 ubiquitin-protein ligase RNF216 |
| SERTAD1 | Q9UHV2 | SERTA domain-containing protein 1 |
| SFTPA1 | Q8IWL2 | Pulmonary surfactant-associated protein A1 |
| SFTPA2 | Q8IWL1 | Pulmonary surfactant-associated protein A2 |
| SFTPD | P35247 | Pulmonary surfactant-associated protein D |
| SIGIRR | Q6IA17 | Single Ig IL-1-related receptor |
| SMAD3 | P84022 | Mothers against decapentaplegic homolog 3 |
| SMPDL3B | Q92485 | Acid sphingomyelinase-like phosphodiesterase 3b |
| SRC | P12931 | Proto-oncogene tyrosine-protein kinase Src |
| STK11IP | Q8N1F8 | Serine/threonine-protein kinase 11-interacting protein |
| STUB1 | Q9UNE7 | E3 ubiquitin-protein ligase CHIP |
| SYK | P43405 | Tyrosine-protein kinase SYK |
| TBK1 | Q9UHD2 | Serine/threonine-protein kinase TBK1 |
| TICAM1 | Q8IUC6 | TIR domain-containing adapter molecule 1 |
| TICAM2 | Q86XR7 | TIR domain-containing adapter molecule 2 |
| TIRAP | P58753 | Toll/interleukin-1 receptor domain-containing adapter protein |
| TLR1 | Q15399 | Toll-like receptor 1 |
| TLR4 | O00206 | Toll-like receptor 4 |
| TLR5 | O60602 | Toll-like receptor 5 |
| TLR6 | Q9Y2C9 | Toll-like receptor 6 |
| TMED7 | Q9Y3B3 | Transmembrane emp24 domain-containing protein 7 |
| TNFSF9 | P41273 | Tumor necrosis factor ligand superfamily member 9 |
| TOLLIP | Q9H0E2 | Toll-interacting protein |
| TRAF3 | Q13114 | TNF receptor-associated factor 3 |
| TRAF6 | Q9Y4K3 | TNF receptor-associated factor 6 |
| TREM1 | Q9NP99 | Triggering receptor expressed on myeloid cells 1 |
| TRIM69 | Q86WT6 | E3 ubiquitin-protein ligase TRIM69 |
| UBQLN1 | Q9UMX0 | Ubiquilin-1 |
| WDFY1 | Q8IWB7 | WD repeat and FYVE domain-containing protein 1 |
